# Metabolic profile associated with distinct behavioral coping strategies of 129Sv and Bl6 mice in repeated motility test

**DOI:** 10.1038/s41598-018-21752-9

**Published:** 2018-02-21

**Authors:** Jane Narvik, Taavi Vanaveski, Jürgen Innos, Mari-Anne Philips, Aigar Ottas, Liina Haring, Mihkel Zilmer, Eero Vasar

**Affiliations:** 1 Department of Physiology, University of Tartu, 19 Ravila Street, Tartu, 50411 Estonia; 2 Department of Biochemistry, Institute of Biomedicine and Translational Medicine, University of Tartu, 19 Ravila Street, Tartu, 50411 Estonia; 3 Center of Excellence for Genomics and Translational Medicine, University of Tartu, 19 Ravila Street, Tartu, 50411 Estonia; 4 Psychiatry Clinic, Tartu University Hospital, 31 Raja Street, Tartu, 50417 Estonia

## Abstract

We investigated the metabolic outcome of different coping strategies in 129S6/SvEvTac (129Sv) and C57BL/6Ntac (Bl6) strains. Two different batches of male 129Sv and Bl6 mice were used. One batch was not subjected to any behavioral manipulations (home cage control; HCC), whereas the other batch was treated with saline for 11 days and exposed after every treatment to the motor activity measurement (repeated motility tested; RMT). Bl6 RMT mice displayed a robust increase in number of rearings during repeated testing. 129Sv RMT mice experienced significant loss of body weight, but showed enhanced weight gain in HCC batch compared to Bl6. Serum metabolites (acylcarnitines, amino acids, biogenic amines, hexoses, glycerophospholipids and sphingolipids) were determined with AbsoluteIDQ p180 kit. Results of the metabolomic study revealed prominent peculiarities between strains in two different conditions. Comparison of both batches of mice demonstrated that in Bl6 biogenic amines (acetyl-ornithine, alpha-amionadipic acid, carnosine) and lysophosphatidylcholine PC(16:1/0:0) dominated. However in 129Sv acylcarnitine C5 clearly dominated, indicating shift towards short-chain acylcarnitines. Stable strain-specific ratios also emerged for both lines, ratio of glycine/PC ae C38:2 for Bl6 and ratios of C5/C0 as well as PC(16:0/0:0)/PC(16:1/0:0) for 129Sv. The described metabolic changes probably reflect different behavioral coping strategies of 129Sv and Bl6 mice.

## Introduction

Bl6 and 129Sv are widely used mouse lines in biomedical research. The classical method of creating transgenic mice is based on these two lines: 129Sv derived mouse embryonic stem cells are applied for introducing targeted mutations into mouse genome^1^, and the Bl6 strain is employed as a background line in transgenic studies^2^. It is commonly accepted that Bl6 mice are more active and venturous, while 129Sv mice are rather idle and display higher level of anxiety^3–6^. Nevertheless, there are no remarkable differences in muscular strength, spatial working memory or in habituation to an open field^3^. However, tests estimating depressive-like behavior demonstrate 129Sv mice to be more vulnerable to stress. Several studies have shown the 129Sv strain to remain more immobile in behavioral despair tests (forced swimming and tail suspension) compared to the Bl6^7,8^, though both strains are often reported to respond similarly to antidepressant drugs interacting with monoaminergic neurotransmission^9–11^. Furthermore, drug-induced hyperactivity is more severe and persistent in Bl6 mice compared to 129Sv, indicating differences in the reactivity and sensitivity of the dopaminergic system in these two mouse lines^12^.

In a recent study, we confirmed that the phenotype differences between the 129Sv and Bl6 strains remain stable in most tests despite environmental modifications^6^. Environmental enrichment and long-term individual housing influences the 129Sv and Bl6 strains differently by reinforcing already existing predispositions in these inbred strains. For example, the activity of Bl6 mice was significantly enhanced during repeated behavioral testing, while 129Sv mice remained inert, but experienced significant loss of body weight^5,6^. Environmental enrichment seems to reinforce existing predispositions in both strains by inducing an active coping strategy in Bl6 and passive coping strategy in 129Sv^6^. These strain differences in the exploratory drive and motivational system of the 129Sv and Bl6 are acknowledged among researchers^4^. Considering behavioral differences and diversity of strain-specific outcomes in pharmacological studies^10,13,14^, we expect to see variation in metabolic profile of these two inbred strains. For this reason we decided to identify possible metabolic consequences of distinct behavioral responses of Bl6 and 129Sv in repeated motility test.

Two batches of male 129Sv and Bl6 mice were used. One batch was used as a home cage control (HCC). This batch was not subjected to any other manipulations than usual routines of the animal house, including the measurement of body weight on the 1^st^ and 11^th^ day. The other batch was treated with saline for 11 days and exposed after every treatment to the motility boxes (repeated motility tested; RMT). In order to measure the metabolite levels of acylcarnitines, amino acids, biogenic amines, level of hexoses, glycerophospholipids and sphingolipids, blood samples were collected from the trunk of the animal immediately after the last behavioral measurement or for home cage controls after taking them directly from cages. Serum was extracted and metabolite levels were determined with the AbsoluteIDQ p180 kit, using a combination of flow injection analysis and liquid chromatography tandem mass-spectrometry technique. We measured 188 metabolites, of which 164 in HCC and 160 metabolites in RMT batch had non-zero values. In HCC 24 acylcarnitines, 35 amino acids and biogenic amines, level of hexoses, 89 glycerophospholipids (13 lysophosphatidylcholine acyls, 38 phosphatidylcholine diacyls, 38 phosphatidylcholine acyl-alkyls), and 15 sphingolipids. In RMT 24 acylcarnitines, 36 amino acids and biogenic amines, level of hexoses, 86 glycerophospholipids (13 lysophosphatidylcholine acyls, 37 phosphatidylcholine diacyls, 36 phosphatidylcholine acyl-alkyls), and 13 sphingolipids. So far, such a comparative study has not been performed in these mouse lines.

## Results

### The body weight changes and metabolic profile of Bl6 and 129Sv in HCCs

**Figure 1:**
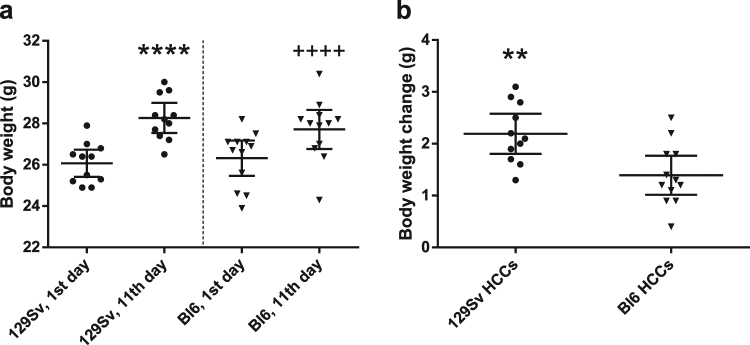
Body weight of 129Sv and Bl6 in HCC batch. Body weight on 1^st^ and 11^th^ day (**a**) and total body weight change during experiment (**b**) for 129Sv and Bl6 in HCCs. 1^st^ vs 11^th^ day revealed weight gain in both strains (****p < 0.0001; ^++++^p < 0.0001). However, the gain of body weight was more pronounced in 129Sv (2.19 ± 0.58 g) compared to Bl6 (1.39 ± 0.59 g; **p = 0.0036; **b**).

The body weight of 129Sv and Bl6 was measured twice: on the 1^st^ day (on the 5^th^ day from arrival) and on the 11^th^ day (on the 15^th^ day from arrival) before collecting blood samples for metabolite measurements. Comparison of body weight on the 1^st^ vs 11^th^ day revealed weight gain in both strains (for 129Sv 26.07 ± 0.98 g vs 28.26 ± 1.08 g; paired t_(10)_ = 12.63, p < 0.0001 and for Bl6 26.32 ± 1.35 g vs 27.71 ± 1.5 g; paired t_(11)_ = 8.15, p < 0.0001; Fig. 1a). However, the gain was more pronounced in 129Sv (2.19 ± 0.58 g) compared to Bl6 (1.39 ± 0.59 g; t_(21)_ = 3.28, p = 0.0036; Fig. 1b). The applied metabolic assay allowed the detection of 164 metabolites (Supplementary Table S1), of which 76 metabolites were significantly different between 129Sv and Bl6 based on Mann-Whitney U test (p ≤ 0.05). After Bonferroni correction 13 metabolites were significantly different in comparison of 129Sv and Bl6 mouse lines; more precisely 5 metabolites showed higher values in Bl6 mice (Table 1) and 8 metabolites were elevated in 129Sv (Table 2).

**Table 1:** Significantly elevated metabolite levels in Bl6 compared to 129Sv in HCCs.

Metabolite	Bl6 (n = 12)	129Sv (n = 10)	Z - score	*p*-value	Eta^2^
	Median (range)	Median (range)			
**Amino acids and biogenic amines**					
Acetyl-ornithine	10.6 (8.88–14.1)	4.53 (3.51–5.39)	3.92	0.00009	0.7
Alpha-aminoadipic acid	10.6 (8.07–14.3)	5.1 (2.74–8.21)	3.86	0.0001	0.68
**Glycerophospholipids**					
***Lysophosphatidylcholine acyls***					
PC(16:1/0:0)	8.59 (6.13–15.9)	3.29 (1.30–5.06)	3.92	0.00009	0.7
PC(20:3/0:0)	11.8 (6.55–18.1)	5.69 (2.23–8.22)	3.66	0.0002	0.61
***Phosphatidylcholine diacyls***					
PC aa C34:3	9.11 (6.78–12.8)	4.95 (3.90–6.95)	3.79	0.0001	0.68

Raw data of marker levels (μM) are presented as median and range. Effect size estimate (Eta^2^) has been calculated by dividing the value of squared standardized test statistic (Z^2^) with the total number of observations (N). From 164 metabolities quantified 5 metabolites remained statistically significant after Bonferroni correction (p ≤ 0.0003) in Bl6 (Mann-Whitney U test non-corrected p-value has been shown). Glycerophospholipids include: lysophosphatidylcholine acyls, phosphatidylcholine diacyls (indicated in italic).

**Table 2:** Significantly elevated metabolite levels in 129Sv compared to Bl6 in HCC.

Metabolite	Bl6 (n = 12)	129Sv (n = 11)	Z - score	*p* - value	Eta^2^
	Median (range)	Median (range)			
**Acylcarnitines**					
C5	0.24 (0.17–0.39)	0.66 (0.38–0.84)	−3.86	0.0001	0.68
**Glycerophospholipids**					
***Phosphatidylcholine acyl-alkyls***					
PC ae C36:2	4.67 (3.08–6.18)	8.73 (6.41–10.2)	−3.92	0.00009	0.7
PC ae C38:2	3.56 (2.27–4.89)	8.31 (5.37–9.90)	−3.92	0.00009	0.7
PC ae C40:4	1.00 (0.63–1.12)	1.49 (1.15–1.70)	−3.92	0.00009	0.7
PC ae C40:6	1.15 (0.78–1.36)	1.75 (1.30–2.08)	−3.76	0.0002	0.64
**Sphingolipids**					
SM (OH) C14:1	0.25 (0.14–0.36)	0.51 (0.37–0.66)	−3.92	0.00009	0.7
SM (OH) C22:1	0.54 (0.40–0.67)	0.85 (0.67–1.02)	−3.92	0.00009	0.7
SM C24:0	2.43 (1.88–2.60)	3.23 (2.77–4.44)	−3.92	0.00009	0.7

Raw data of marker levels (μM) are presented as median and range. Effect size estimate (Eta^2^) has been calculated by dividing the value of squared standardized test statistic (Z^2^) with the total number of observations (N). From 164 metabolities quantified 8 metabolites were statistically significant after Bonferroni correction (p ≤ 0.0003) in 129Sv (Mann-Whitney U test non-corrected p-value has been shown). Glycerophospholipids include: Phosphatidylcholine acyl-alkyls (indicated in italic).

### Metabolites elevated in Bl6 HCCs

Acetyl-ornithine and lysoPC (16:1/0:0) both (Z = 3.92, Eta^2^ = 0.7) displayed most significant elevation in Bl6 compared to 129Sv (Table 1). Significant elevations (Z value > 3, Eta^2^ ≥ 0.61) were also established for biogenic amine alpha-aminoadipic acid, glycerophospholipids PC(20:3/0:0) and PC aa C34:3 (Table 1). Furthermore, ratio of C4/C5 (Z = 3.92, Eta^2^ = 0.7) and several calculated ratios of glycine, including glycine/PC ae 38:2 (Z = 3.92, Eta^2^ = 0.7) and glycine/serine (Z = 3.86, Eta^2^ = 0.68) were elevated in Bl6 (Supplementary Table S3). All above mentioned comparisons survived Bonferroni correction for multiple comparisons (p ≤ 0.0003).

### Metabolites elevated in 129Sv HCCs

Several phosphatidylcholine acyl-alkyls (PC ae C36:2, PC ae C38:2, PC ae C40:4) and sphingolipids (SM (OH) C14:1, SM (OH) C22:1, SM C24:0) displayed the strongest elevation in 129Sv compared to Bl6 (all Z = 3.92, all Eta^2^ = 0.7; Table 2). Significant elevations in the 129Sv were established for acylcarnitine C5 (Z value = 3.76, Eta^2^ = 0.64) and glycerophospholipid PC ae C40:6 (Z value = 3.86, Eta^2^ = 0.68; Table 2). The ratio between PC(16:0/0:0)/PC(16:1/0:0), spermidine/putrescine and C5/C0 were significantly higher in 129Sv compared to Bl6 (all Z ≥ −3.86, Eta^2^ ≥ 0.68; Supplementary Table S4). All above mentioned comparisons survived Bonferroni correction for multiple comparisons (p ≤ 0.0003).

### Metabolite differences highlighted by GLM in HCCs

Using GLM, we confirmed a significant main effect (F_(6, 15)_ = 33.91, partial Eta^2^ = 0.99) of mouse strain on the levels of acylcarnitine C5, glycerophospholipids (PC ae C36:2, PC ae C38:2, PC ae C40:4, PC ae C40:6), sphingolipids (SM (OH) C14:1, SM (OH) C22:1, SM C24:0), biogenic amines (acetyl-ornithine, alpha-aminoadipic acid, carnosine), glycerophospholipids [PC (16:1/0:0), PC(20:3/0:0), PC aa C34:3] and on body weight change in HCC condition (Table 3).

**Table 3:** Regression coefficients (ß), confidence intervals (CI) and significance values of log_10_-transformed metabolite levels adjusted for strain in HCC.

Bl6 and 129Sv comparison	ß	ß (95% CI)	*t - value*	*p* - value
**Acylcarnitines**				
C5	−0,87	(−1.10, −0.65)	−8.02	<0.0000001
**Biogenic amines**				
Acetyl-ornithine	0.95	(0.82, 1.09)	14.36	<0.00000001
Alpha-aminoadipic acid	0.84	(0.58, 1.09)	6.87	0.000001
Carnosine	0.74	(0.43, 1.05)	4.91	0.00008
**Glycerophospholipids**				
***Lysophosphatidylcholine acyls***				
PC(16:1/0:0)	0.84	(0.59, 1.09)	6.99	0.000001
PC(20:3/0:0)	0.76	(0.46, 1.06)	5.23	<0.0001
***Phosphatidylcholine diacyl***				
PC-aa-C34:3	0.84	(0.59, 1.09)	6.99	0.000001
***Phosphatidylcholine acyl-alkyls***				
PC-ae-C36:2	−0.86	(−1.10, −0.62)	−7.50	<0.000001
PC-ae-C38:2	−0.89	(−1.10, −0.68)	−8.91	<0.0000001
PC-ae-C40:4	−0.82	(−1.09, −0.55)	−6.37	<0.00001
PC-ae-C40:6	−0.79	(−1.07, −0.50)	−5.71	<0.00001
**Sphingolipids**				
SM-(OH)-C14:1	−0.83	(−1.09, −0.57)	−6.65	<0.00001
SM-(OH)-C22:1	−0.86	(−1.10, −0.61)	−7.39	<0.000001
SM-C24:0	−0.82	(−1.09, −0.55)	−6.40	<0.00001
**Behavioral parameter**				
Change in body weight	−0.55	(−0.94–0.16)	−2.96	0.01

F_(6,15)_ = 33.91, p = 0.0002, partial Eta^2^ = 0.99. Glycerophospholipids include: lysophosphatidylcholine acyls, phosphatidylcholine diacyls, and phosphatidylcholine acyl-alkyls (indicated in italic).

### The behavioral and body weight changes of 129Sv and Bl6 in RMT mice

**Figure 2:**
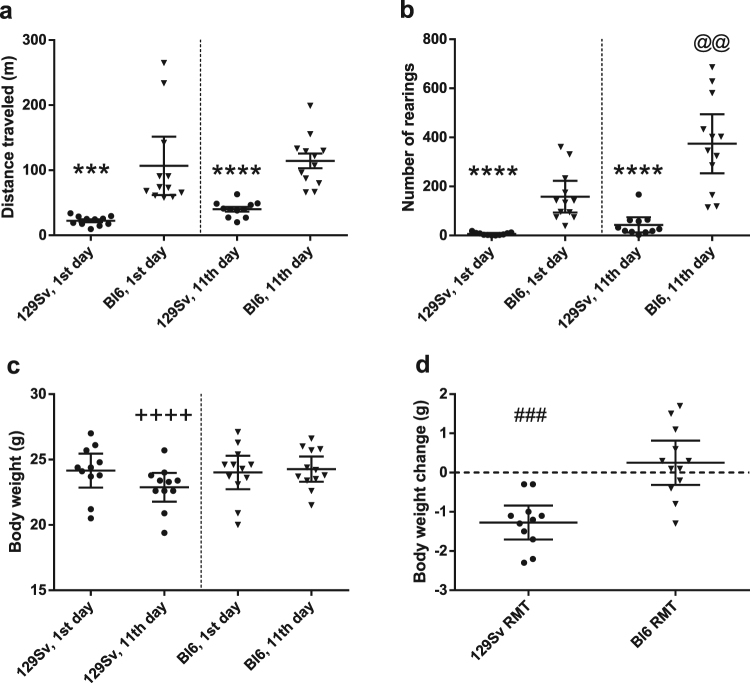
Motility and body weight of 129Sv and Bl6 in RMT batch. Main effects of distance travelled (**a**), number of rearings (**b**), body weight (**c**) and body weight change (**d**). T-test was applied to demostrate differences between 1^st^ and 11^th^ day. ***p < 0.001 and ****p < 0.00001 indicate differences between strains on corresponding days (**a**,**b**). ^@@^p = 0.005 indicates difference between 1^st^ and 11^th^ day of rearings in Bl6 (**b**). ^++++^p < 0.00001 indicates absolute weight on 1^st^ and 11^th^ day (**c**) and ^###^p < 0.0001 weight change in 129Sv HCC batch during experiment (**d**). After repeated manipulation, modest elevation of body weight was seen in Bl6 (0.25 ± 0.26) and significant reduction of body weight was established in 129Sv (−1.27 ± 0.20; ^###^p = 0.0001; **d**). More information about repeated testing can be found in the Supplementary Fig. S1.

As expected, Bl6 and 129Sv displayed significantly different motor behavior. Repeated measures ANOVA revealed a statistically significant strain effect for distance travelled (strain effect F_(1,21)_ = 41.52; p = 0.000002; repeated experiments F_(1,21)_ = 1.04; p = 0.32; strain × repeated experiments F_(1,21)_ = 0.16; p = 0.69). Distance travelled on day 1 by Bl6 was significantly longer compared to 129Sv (t_(21)_ = 3.93; p = 0.0008). This difference remained statistically significant on day 11 as well (t_(21)_ = 6.07; p = 0.000005; Fig. 2a; Supplementary Fig. S1a). The frequency of rearings was also strongly in favor of Bl6. The initial difference in vertical activity between the strains increased during repeated testing (strain: F_(1,21)_ = 51.51, p = 0.0000001; repeated experiments F_(1,21)_ = 14.84, p = 0.0009; strain × repeated experiments F_(1,21)_ = 7,41, p = 0.013). On day 1, Bl6 performed more rearings compared to 129Sv (t_(21)_ = 4.93; 0.00007; Fig. 2b; Supplementary Fig. S1b) and by day 11 the difference had further increased (t_(21)_ = 5.62; p = 0.00001). In Bl6 the frequency of rearings was elevated more than two-fold by repeated testing (158 ± 102 on 1^st^ *vs*. 374 ± 190 on 11^th^ day; paired t_(11)_ = −3.51; p = 0.005). Body weight measurements also showed a significant difference between the two strains after repeated testing (repeated measures ANOVA; strain effect F_(1,21)_ = 0.73, p = 0.40; repeated experiments F_(1,21)_ = 9.79, p = 0.005; strain effect × repeated experiments F_(1,21)_ = 21.71, p = 0.0001; Supplementary Fig. S1c). In the beginning of the behavioral experiment both strains had nearly identical body weight. Comparison of body weight on the 1^st^ vs 11^th^ day revealed weight loss in 129Sv (24.15 ± 1.93 g vs 22.88 ± 1.64 g; paired t_(10)_ = 6,53, p < 0.0001) and stabilization in Bl6 (24.02 ± 2.02 g vs 24.27 ± 1.51 g; paired t_(11)_ = 0.97, p = 0.3508; Fig. 2c). After repeated manipulations, marginal increase was seen in Bl6 (0.25 ± 0.26) and significant reduction of body weight was established for 129Sv (−1.27 ± 0.20; t_(21)_ = 4.66, p = 0.0001; Fig. 2d).

### Metabolic profile of Bl6 and 129Sv in RMT

The applied metabolic assay allowed the detection of 160 metabolites (Supplementary Table S5), of which 52 metabolites were significantly different between 129Sv and Bl6 mice. After Bonferroni correction 5 metabolites were significantly different between 129Sv and Bl6 in RMT batch; 4 metabolites showed higher values in Bl6 and 1 metabolite survived Bonferroni correction in 129Sv (Table 4).

**Table 4:** Significantly elevated metabolite levels for both strains in RMT.

Metabolite	Bl6 (n = 12)	129Sv (n = 11)	Z - score	*p*-value	Eta^2^
	Median (range)	Median (range)			
**Significantly elevated metabolite levels in Bl6**					
**Amino acids and biogenic amines**					
Acetyl-ornithine	15.9 (10.6–19.1)	7.25 (5.40–12.1)	3.91	0.00009	0.67
Alpha-aminoadipic acid	10.950 (7.420–17.200)	0.000 (0.000–9.490)	3.85	0.0001	0.65
Carnosine	15.6 (3.20–21.2)	2.79 (1.17–7.34)	3.72	0.0002	0.60
**Glycerophospholipids**					
***Lysophosphatidylcholine acyls***					
PC(16:1/0:0)	12.8 (6.31–17.8)	5.87 (3.50–8.26)	3.79	0.0002	0.62
**Significantly elevated metabolite levels in 129**Sv					
**Acylcarnitines**					
C5	0.22 (0.18–0.28)	0.40 (0.23–0.63)	−3.79	0.0002	0.63

Raw data of marker levels (μM) are presented as median and range. Effect size estimate (Eta^2^) has been calculated by dividing the value of squared standardized test statistic (Z^2^) with the total number of observations (N). After application of Bonferroni correction (p ≤ 0.0003) 5 metabolites remained statistically significant in comparison of 129Sv and Bl6 in RMT batch (Mann-Whitney U test non-corrected p-value has been shown); 4 metabolites in Bl6 and one metabolite in 129Sv. Glycerophospholipids include: lysophosphatidylcholine acyls (indicated in italic).

### Metabolites elevated in Bl6 RMT mice

Acetyl-ornithine displayed the most significant elevation in Bl6 compared to 129Sv (Z = 3.91, Eta^2^ = 0.67; Table 4). Significant elevations (Z value ≥ 3.72, Eta^2^ ≥ 0.60) were also established for biogenic amines (alpha-aminoadipic acid, carnosine), glycerophospholipid PC(16:1/0:0), the ratios of glycine/PC ae 38:2 and C3/C4 (Z value ≥ 3.76, Eta^2^ ≥ 0.61; Table 4). All above mentioned comparisons survived Bonferroni correction for multiple comparisons (p ≤ 0.0003).

### Metabolites elevated in 129Sv RMT mice

Significant elevation (Z value > 3, Eta^2^ ≥ 0.4) in the 129Sv group was established for acylcarnitine C5 (Table 4). The following ratios were also significant (Z value ≥ 3.60, Eta^2^ ≥ 0.56): Fisher ratio, C4/C0, C5/C0, C14/C16:1, C16/C16:1, C18/C18:1 and PC(16:0/0:0)/PC(16:1/0:0) (Supplementary Table S6 and S8). All above mentioned comparisons survived Bonferroni correction for multiple comparisons (p ≤ 0.0003).

### Metabolite differences highlighted by GLM in RMT mice

Altogether 5 metabolites survived Bonferroni correction for multiple comparisons and were included into GLM test. These metabolites [acylcarnitine C5, acetyl-ornithine, alpha-aminoadipic acid, carnosine and PC(16:1/0:0)] as well as measured behavioral parameters (distance travelled, number of rearings, changes in body weight) accounted for 99% (F_(8,8)_ = 143.5, p = 0.0000001) of the mouse strain differences in RMT condition (Table 5). Thus, our results indicate that there is a strong strain-dependent (Bl6 *vs*. 129Sv) interplay among metabolic markers and behavioral characteristics in both (HCC, RMT) condition.

**Table 5:** Regression coefficients (ß), confidence intervals (CI) and significance values of log_10_-transformed metabolite levels adjusted for strain in RMT.

Bl6 and 129Sv comparison	ß	ß (95% CI)	*t - value*	*p* - value
**Acylcarnitine**				
C5	−0.88	(−1.14, −0.61)	−7.10	<0.00001
**Amino acids and biogenic amines**				
Acetyl-ornithine	0.81	(0.49, 1.13)	5.38	<0.0001
Alpha-aminoadipic acid	0.74	(0.36, 1.11)	4.21	<0.001
Carnosine	0.75	(0.39, 1.11)	4.40	<0.001
**Glycerophospholipids**				
***Lysophosphatidylcholine acyl***				
PC(16:1/0:0)	0.71	(0.32, 1.10)	3.88	<0.01
**Behavioral parameters**				
Distance travelled	0.81	(0.49, 1.13)	5.42	<0.0001
Number of rearings	0.75	(0.38, 1.11)	4.35	<0.001
Change in body weight	0.63	(0.20, 1.06)	3.15	<0.01

Glycerophospholipids include: lysophosphatidylcholine acyls (indicated in italic). F_(8,8)_ = 143.5, p = 0.0000001, partial Eta^2^ = 0.99. Glycerophospholipids include: lysophosphatidylcholine acyls (indicated in italic).

## Discussion

### Behavioral and body weight differences in Bl6 and 129Sv

Comparison of Bl6 and 129Sv in HCC batch demonstrated that during the 11-day follow-up period the body weight gain of 129Sv (2.15 ± 0.17 grams) was more pronounced compared to Bl6 (1.39 ± 0.17 grams). It is also commonly accepted that Bl6 are more active and venturous, while 129Sv are quite idle and often more anxious^3–6^. Therefore, longer distance travelled and higher number of rearings in RMT were expected for Bl6. After repeated exposure to the motility boxes, the frequency of rearings in Bl6 increased robustly, most likely reflecting a significant increase in the exploratory drive. By contrast, the locomotor activity of 129Sv was not markedly affected by RMT. However, the 129Sv responded differently in RMT compared to HCC, with a significant reduction of body weight, a change not seen in Bl6. The same effect has been shown after the exposure of these mouse lines to environmental enrichment^5,6^. Hence, it is apparent that these two mouse lines display distinct behavioral strategies. RMT reinforced the predisposition in both strains, by evoking an active coping strategy in Bl6, while 129Sv developed a more passive strategy or even aversion towards the test situation.

One should keep in mind that the 129Sv and all related 129 strains carry a 25 bp frameshift deletion within exon 6 of the *Disc1* gene resulting in a premature termination codon at exon 7^15^. Koike *et al*.^16^ discovered the deletion while modifying the 129Sv *Disc1* allele to imitate the production of the hypothetical C-terminally truncated protein product. Moreover, they reported a significant difference in a delayed non-match to place test, a specific test of working memory, that was significantly altered in both 129Sv *Disc1* heterozygotes and homozygotes compared to Bl6^16^. Recent evidence suggests a prominent role of *DISC1* gene in the genetics of major psychiatric disorders like schizophrenia, bipolar and major depressive disorder^17^. The studies in rats demonstrate that misassembly of full-length DISC1 protein alters dopamine homeostasis, leading to apparent behavioral deficits^18^. Indirect evidence supporting the reduced activity of the dopaminergic system in 129Sv comes from the measurement of tyrosine, the precursor molecule of dopamine and noradrenaline. Tyrosine levels displayed higher tendency in Bl6 RMT batch compared to HCC. Moreover, the ratio of tyrosine and phenylalanine also demonstrated a higher tendency in Bl6 both batches (Supplementary Tables S3 and S7). Both of these findings reflect the likelyhood of higher catecholamine precursor availability in Bl6. Altogether, the dysfunction of DISC1 and dopamine system may explain the aberrant response of 129Sv mice to the environmental enrichment^6^ as well as to the repeated exposure described in this study. However, this hypothesis needs further validation.

### Metabolic profile and behavioral response of mouse strains

#### Acylcarnitines and hexoses

Metabolomic study was performed with serum samples collected from HCC batch after weighing and in the case of RMT batch immediately after the last exposure of mice to the motility boxes. Both strains revealed rather distinct profiles of acylcarnitines and hexoses. The level of hexoses was higher in Bl6 for both batches compared to 129Sv, but these comparisons did not survive Bonferroni correction (Supplementary Tables S1 and S5). In both batches of 129Sv acetylcarnitine C5 and ratio of C5/C0 remained stable markers after Bonferroni correction (Tables 2, 4 and 6), while the ratios of C16.0/C16.1 and C18.0/C18.1 in comparison of 129Sv HCC and RMT batch changed remarkably (Table 7). In RMT batch 129Sv lost body weight probably due to compromised food motivation caused by the repeated testing. There is evidence from a rat study that reduced food intake decreases the level of carnitine (C0), but increases the levels of short-chain acylcarnitines^19^. In current study, a similar metabolic shift between acylcarnitines (C4, C5) and carnitine C0 occurred in RMT animals (Supplementary Table S8). One may suggest that the repeated behavioral testing was more stressful for 129Sv than Bl6. The reason for elevation of hexoses in Bl6 compared to 129Sv is not clear and remains to be clarified in further studies.

**Table 6:** List of stable metabolites and ratios in Bl6 and 129Sv respectively.

Metabolites	Bl6 mice (Eta^2^ values)		Metabolites	129Sv mice (Eta^2^ values)	
	Home cage	Repeatedly tested		Home cage	Repeatedly tested
Acetyl-ornithine	0.70	0.67	C5	0.68	0.63
PC(16:1/0:0)	0.70	0.63			
Alpha-aminoadipic acid	0.68	0.64			
Carnosine	0.57	0.60			
**Ratios**			**Ratios**		
Glycine/PC ae C38:2	0.70	0.69	C5/C0	0.69	0.72
			PC(16:0/0:0)/PC(16:1/0:0)	0.70	0.71

Effect size (Eta^2^) estimates for the Mann-Whitney U tests. Effect size estimate (Eta^2^) has been calculated by dividing the value of squared standardized test statistic (Z^2^) with the total number of observations (N).

**Table 7:** List of metabolites and ratios undergoing significant change in Bl6 and 129Sv due to repeated behavioral testing.

Metabolites	Bl6 mice (Eta^2^ values)		Metabolites	129Sv mice (Eta^2^ values)	
	Home cage	Repeatedly tested		Home cage	Repeatedly tested
PC(20:3/0:0)	**0.61**	0.32	PC ae C36:2	**0.70**	0.41
PC(18:1/0:0)	**0.57**	0.22	SM (OH) C14:1	**0.70**	0.41
C4/C5*	**0.70**	0.21	SM (OH) C22:1	**0.70**	0.4
Glycine/serine*	**0.68**	0.31	SM C24:0	**0.70**	0.23
PC aa C32:1	0.32	**0.49**	Fisher ratio*	0.39	**0.71**
PC aa C34:4	0.32	**0.51**	C16.0/C16.1*	0.26	**0.58**
Hexoses	0.31	**0.49**	C18.0/C18.1*	0.23	**0.69**

Effect size (Eta^2^) estimates for the Mann-Whitney U tests. Effect size estimate has been calculated by dividing the value of squared standardized test statistic (Z^2^) with the total number of observations (N). Ratios have been indicated by*.

#### Amino acids and biogenic amines

In both batches of Bl6 the levels of biogenic amines (acetyl-ornithine, alpha-aminoadipic acid, carnosine) were significantly higher compared to 129Sv. Definitely, acetyl-ornithine, alpha-aminoadipic acid and carnosine belong to the metabolic signatures of Bl6. Dipeptide carnosine (*β*-alanyl-L-histidine) is highly concentrated in the muscle and brain. It acts as an antiglycating agent, reducing the formation rate of advanced glycation end-products, and may act as a neuroprotective mediator^20^. Alpha-aminoadipic acid is a component of lysine (Lys) metabolism pathway and a marker of oxidative stress^21,22^. A recent metabolomic study of diabetes patients plasma samples suggested that alpha-aminoadipic acid may be a modulator of glucose homeostasis and diabetes risk^23^. Studies in rodents have also shown that alpha-aminoadipic acid modulates kynurenic acid levels in the brain. Kynurenic acid is a neuroactive metabolite that interacts with NMDA, AMPA/kainate and alpha 7 nicotinic receptors^24^. In experiments with rat brain tissue slices, alpha-aminoadipic acid exposure resulted in a substantial decrease in levels of kynurenic acid^25^. Similarly, *in vivo* studies in free-moving rats exposed to alpha-aminoadipic acid through microdialysis in the hippocampus resulted in a robust decrease in kynurenic acid level^26^. Alpha-aminoadipic acid is a substrate of the enzyme alpha-aminoadipic acid aminotransferase II, which has been shown to be the same enzyme as kynurenine aminotransferase II (KAT-II), and is responsible for the transamination of L - kynurenine to kynurenic acid^27,28^. Alpha-aminoadipic acid levels dictate the availability of KAT-II for the transamination of L–kynurenine to kynurenic acid^29^.

The ratio of BCAA/AAA or Fisher ratio was higher in both batches of 129Sv (Supplementary Tables S2 and S6). Fisher ratio was a marker that survived Bonferroni correction in 129Sv RMT batch. The ratios of short-chain acylcarnitines (C4, C5) to carnitine (C0) were higher in 129Sv (Supplementary Table S8). There is evidence that short-chain acylcarnitines (C3, C4, C5) are formed from BCAAs^30^. Isoleucine and leucine play a role in the formation of C5^30^, showing an apparent link between amino acid and energy metabolism. This is in line with the increased level of acylcarnitine C5 in our study. It is possible that C5 as well as its ratio with carnitine and augmented BCAA levels reflect the changes in energy metabolism of 129Sv compared to Bl6.

#### Lysophosphatidylcholines (LysoPCs)

Only the increased values of lysoPC(16:1/0:0) in both batches of Bl6 strain and ratio of PC(16:0/0:0)/PC(16:1/0:0) in both batches of 129Sv strain survived Bonferroni correction (Supplementary Tables S4 and S8). LysoPCs are bioactive pro-inflammatory lipids generated by pathological activities^31^. LysoPCs up-regulate the expression of inflammation-related genes *IL-6*, *TNF-α*, *Ccl5*, *Cxcl1*, and *iNOS*^32^. It has been demonstrated that LysoPCs, particularly PC(16:0/0:0) increase the formation of IFN-γ in human T lymphocytes^33,34^. Nevertheless, the functional role of established differences between LysoPC in 129Sv and Bl6 is not clear and remains to be established in the further studies.

#### Phosphatidylcholines (PCs)

Among HCCs the elevation of PCs was more prominent in 129Sv than in Bl6 (Table 2). In Bl6 only PC aa C34:3 (Eta^2^ = 0.68) survived Bonferroni correction (Table 1). In 129Sv HCC 4 PC acyl-alkyls were elevated compared to Bl6. Prominent elevations were established for these four PC acyl-alkyls (PC ae C36:2, PC ae C38:2, PC ae C40:4, Eta^2^ = 0.7 for all three) and PC ae C40:6 (Eta^2^ = 0.64) in 129Sv (Table 2). The stronger elevation of PCs in 129Sv HCC may be linked to the higher body weight gain in these mice, possibly indicating elevated lipid metabolism. In RMT these changes were less variable in both strains. In Bl6 only one PC diacyls was elevated (Table 4), whereas all PCs in 129Sv did not survive Bonferroni correction. The outcome of Bonferroni correction was supported by GLM. Listed PC acyl-alkyls (PC ae C36:2, PC ae C38:2, PC ae C40:4, PC ae C40:6) in HCC were positively associated with body weight gain in 129Sv (Table 3).

#### Sphingolipids

In the 129Sv HCC batch 3 sphingolipids SM (OH) C14:1, SM (OH) C22:1 and SM C24:0 (Eta^2^ = 0.7) survived Bonferroni correction, while none of sphingolipids survived Bonferroni correction in 129Sv RMT batch (Tables 2 and 7). GLM established association between four sphingolipids [SM (OH) C14:1, SM (OH) C22:1, SM (OH) C22:2 and SM C24:0] and elevated body weight in 129Sv HCC batch (Table 3). Sphingolipids are one of the major lipid components of eukaryotic membranes and have a wide range of physiological functions, including cell adhesion, skin permeability barrier formation, myelin maintenance, immunity, spermatogenesis and glucose metabolism^35,36^. Complex sphingolipids located in the plasma membrane of animal cells, especially nerve cells, have a structural function and are believed to protect the cell surface from harmful environmental factors. They also serve as adhesion sites for extracellular proteins, play important roles in signal transmission, and cell recognition^37^. The elevated levels of sphingolipids in HCC could reflect increased lipid metabolism in 129Sv. In RMT animals the balance of sphingolipids still favors 129Sv, but the increase is less prominent compared to HCCs. The recent evidence suggests that the decline of several PCs and sphingolipids impairs the liver-dependent lipid metabolism and circulation, as hepatic PCs are required for the assembly and secretion of very low-density lipoprotein from the liver^38,39^.

#### Impact of repeated testing on metabolite levels

Our analysis demonstrated that the differences of certain metabolites in comparison of both batches (HCC and RMT) of Bl6 and 129Sv remained unchanged (Supplementary Table S9). After Bonferroni correction and application of GLM the following metabolites remained similarly elevated in both batches of Bl6: biogenic amines (acetyl-ornithine, alpha-aminoadipic acid, carnosine), lysophoshatidylcholine PC(16:1/0:0) and the increased ratio of glycine/PC ae C38:2 (Table 6). In both batches of 129Sv the elevation of only one metabolite remained unchanged: acylcarnitine C5. Also, the ratio of acylcarnitine C5/C0 and PC(16:0/0:0)/PC(16:1/0:0) demonstrated a stable elevation in both batches of 129Sv (Table 6). One may suggest that these stable differences in metabolite levels of 129Sv and Bl6 reflect their strain-specific metabolic signatures. Several molecules also undergo a significant change in Bl6 and 129Sv under the influence of RMT. The effect size of lysophosphatidylcholines PC(18:1/0:0), PC(20:3/0:0), as well as ratio of C4/C5 and glycine/serine were reduced in Bl6 RMT batch (Table 7). In 129Sv RMT the reduction of effect sizes was evident for PC ae C36:2 and for several sphingolipids (SM C24:0, SM (OH) C14:1, SM (OH) C22:1). In 129Sv RMT increased the effect size for Fisher ratio, indicating a shift towards BCAAs over AAAs (Table 7).

## Conclusions

After repeated exposure to the motility boxes, the frequency of rearings increased robustly in Bl6, most likely reflecting a significant increase in the exploratory drive. By contrast, the low exploratory activity of 129Sv was not significantly affected by RMT. However, 129Sv responded differently in RMT compared to HCC batch, with a significant reduction of body weight, a change not established in Bl6. Interestingly, 129Sv showed more pronounced weight gain in HCC batch compared to Bl6. Hence, it is apparent that these two mouse lines display distinct behavioral coping strategies. RMT reinforced the coping predisposition in both strains, by evoking an active coping strategy in Bl6, while a more passive strategy developed in 129Sv strain. Besides, these mouse strains display apparent differences in their metabolic profile. The metabolites most significantly elevated in Bl6 (both in HCC and RMT) include biogenic amines (acetyl-ornithine, alpha-aminoadipic acid, carnosine) and lysophosphtidylcholine PC(16:1/0:0). In 129Sv one metabolite clearly dominates – acylcarnitine C5. The elevated levels of short-chain acylcarnitine C5 and its ratio to carnitine in 129Sv RMT probably indicates reduced food intake. However, the role of above mentioned metabolites in different behavioral coping strategies of two mice strains is not clear and remains to be established in further studies.

## Materials and Methods

### Animals

Two batches of male 129Sv and Bl6 mice were used in this study. One batch of these two inbred lines (C57BL/6NTac; Taconic Germantown, New York; n = 12 and 129S6/SvEvTac; Taconic Germantown, New York; n = 10) was used as home cage controls (HCCs). After the arrival from breeder the mice were habituated for 15 days before the blood sampling. At the time of sample collection animals were on average 10 weeks old. The other batch (C57BL/6NTac; Taconic Germantown, New York; n = 12 and 129S6/SvEvTac; Taconic Germantown, New York; n = 11) was subjected to repeated motility testing (RMT batch). These animals were bred in the local animal facility and were weaned from the mother at the age of 3 weeks, thereafter divided into home cages with up to 10 pups. The animals were housed under a 12 h light/dark cycle with lights on at 7:00 a.m. Animals were housed in their respective home cages (1290D Eurostandard type III cages; 425 × 276 × 153 mm; Tecniplast, Italy) with bedding and nesting material. The bedding (aspen chips) and nesting material (aspen wool) were changed weekly. The animals had *ad libitum* access to Ssniff universal mouse and rat maintenance diet (cat# V1534; Ssniff, Germany) and reverse osmosis-purified water, except for 1 hour during testing in the RMT batch. Behavioral testing, including habituation, started at the age of 6–9 weeks, and lasted for 13 days. At the time of sample collection, animals were on average 10 weeks old.

### Behavioral testing

HCCs were weighed twice: on the 5^th^ day (the 1^st^ day) from arrival and on the 15^th^ day (the 11^th^ day), right before taking blood samples. The RMT batch was allocated for behavioral testing for a period of 13 days. The first two days were used for adaptation to the testing environment, followed by experimental days 3–13 (hereinafter days 1–11) for locomotor activity measurements. On test days 1–11 the following routine was used: animals were weighed, 0.9% saline solution was administered i.p. in volume of 10 ml/kg and animals were placed for 30 min into single housing cages (1284 L Eurostandard type II cages, 425 × 276 × 153 mm, Tecniplast, Italy). After 30 min of single housing, animals were placed into the motility boxes for 30 min locomotor activity measurement and then returned to home-cages. This test was conducted in a lit room (around 400 ± 25 lx) in soundproof photoelectric motility boxes (448 × 448 × 450 mm) made of transparent Plexiglas and connected to a computer (TSE Technical & Scientific Equipment GmbH, Germany). After each mouse the floor of boxes was cleaned with 5% ethanol solution. Software registered the distance travelled and number of rearings. Latin square design was used to randomize daily measurement cycles. On day 11, after the locomotor activity recordings, animals were killed by cervical dislocation, decapitated and trunk blood was collected for the metabolomic analysis. Eleven-day follow-up period was chosen because during this time period all the established behavioral and body weight changes were more or less stabilized (Supplementary Fig. S1a–c).

### Sample collection

Blood sampling tubes were pre-processed with 20 µl of EDTA (ethylenediaminetetraacetic acid). Tubes with blood samples were shaken and kept at room temperature for about 30 minutes, followed by centrifugation at 2000 × g for 15 min in 4 °C. Serum was placed into new tubes and stored at −80 °C until use^40^.

### Measurement of metabolites in serum samples

The endogenous metabolites were analyzed with AbsoluteID p180 Kit (Biocrates Life Sciences AG, Innsbruck, Austria). We measured 188 metabolites, of which 164 in HCC and 160 metabolites in RMT batch had non-zero values. This validated assay allows comprehensive identification and quantification of amino acids, acylcarnitines, biogenic amines, hexoses, phospho- and sphingolipids (phosphatidylcholines, lysophosphatidylcholines, sphingomyelins). Analyzed glycerophospholipids (lysophosphatidylcholines, phosphatidylcholines) are differentiated according to the presence of ester and ether bonds in the glycerol moiety. The “aa” indicates that fatty acids at the sn-1 and the sn-2 position are bound to the glycerol backbone via ester bonds, while “ae” denotes that fatty acid at the sn-1 position are bound via ether bond. The total number of carbon atoms and double bonds present in lipid’s fatty acid chains are denoted as “C x: y,” where x indicates the number of carbons and y the number of double bonds. Serum levels of metabolites were determined using a flow injection analysis tandem mass-spectrometry (FIA-MS/MS) as well as a liquid chromatography mass-spectrometry (LC-MS/MS) technique on a QTRAP 4500 mass-spectrometer (Sciex, USA). All preparations and measurements were performed as described in the manufacturer’s kit manual. Identification and quantification of the metabolites were achieved using multiple reaction monitoring (MRM) along with internal standards. Data quality was checked based both on the level of detection and the level of quantification (see also quality control data in Supplementary Table S10). Calculations of metabolite concentrations were automatically performed by MetIDQ software (Biocrates Life Sciences AG, Innsbruck, Austria).

### Statistical analyses

Both batches of male HCC and RMT mice were analyzed separately (in both batches: Bl6 vs 129Sv). The reason for this was the different source of animals and the delayed onset of home cage control study (spring 2016 vs summer 2017), therefore there might be minor differences in preparation of study samples. Shapiro-Wilk test was applied to test for the normality assumption of data. The behavioral and body weight outcomes corresponded to the normal distribution and were analyzed by repeated measures ANOVA (genotype × days 1 and 11), followed by Bonferroni *post hoc* test in RMT or by paired T-test for HCCs. To demonstrate the difference in activity and body weight gain in different strains during experimental period T-test was applied. All statistical tests were two-sided, and only p ≤ 0.05 was considered to be statistically significant. Mean of measurements is shown as mean ± SD. Overall, data on figures is shown as mean ± 95% CI. To compare metabolomic profiles of Bl6 and 129Sv mice in both experimental control condition, we used Mann-Whitney U-test, as the majority of metabolite data did not follow normal distribution. Statistical adjustment for multiple test (Bonferroni correction) was applied for the number of measured biomarkers (164 for HCC and 160 for RMT batch) within particular analysis^41^, and differences between groups were considered significant at p ≤ 0.0003. In addition, to provide an overview about the magnitude of the differences between groups, effect size estimates (Eta^2^) for non-parametric tests were calculated (the value of squared standardized test statistic (Z) was divided by the total number of animals; N = 22 for HCC and N = 23 for RMT). Eta^2^ values of ≥0.14 were defined as large effect^42^. Next, to demonstrate mouse strain dependent main effects on biomarker levels, general linear model (GLM) was applied. Only subsets of biomarkers selected based on the correction for multiple comparison were inputted into GLM. Biomarker values for GLM were log_10_-transformed to satisfy the normality assumption of data. F-tests were used to further compare the fit of linear models and analyze significant main effects in the final models and partial Eta^2^ values (the proportion of the effect in addition to error variance that is attributable to the effect) were established for the final models. Partial Eta^2^ values of ≥0.26 were defined as large effects. All figures were generated by using GraphPad software 7^th^ edition (GraphPad Software, California, USA). All the statistical analyses were performed using Statistica software 13th edition (StatSoft, Oklahoma, USA).

### Ethics

All animal procedures in this study were performed in accordance with the European Communities Directive (2010/63/EU) and permit (No. 29, April 28, 2014) from the Estonian National Board of Animal Experiments. In addition, the use of mice was conducted in accordance to the regulations and guidelines approved by the Laboratory Animal Centre at the Institute of Biomedicine and Translational Medicine.

### Data availability

The datasets generated during and/or analyzed during the current study are available from the corresponding author on reasonable request.

## Electronic supplementary material

Supplementary Information
