# Supplementary material for: Metabolic profile associated with distinct behavioral coping strategies of 129Sv and Bl6 mice in repeated motility test
Source: Sci Rep. 2018 Feb 21;8:3405. doi: 10.1038/s41598-018-21752-9 (PMC5821849; doi:10.1038/s41598-018-21752-9)
Supplement: Supplementary file 1 — Supplementary Information [file 41598_2018_21752_MOESM1_ESM.pdf]

Supplementary material

Metabolic profile associated with distinct behavioral coping strategies of 129Sv and Bl6 mice in repeated motility test

Jane Narvik[a,c], Taavi Vanaveski[a,c]*, Jürgen Innos[a,c], Mari-Anne Philips[a,c], Aigar Ottas[b,c], Liina Haring[c,d], Mihkel Zilmer[b,c], Eero Vasar[a,c]

* Corresponding author
E-mail address: taavi.vanaveski@ut.ee

[a]Department of Physiology, [b]Department of Biochemistry, Institute of Biomedicine and Translational Medicine, University of Tartu, 19 Ravila Street, Tartu 50411, Estonia

[c]Center of Excellence for Genomics and Translational Medicine, University of Tartu, 19 Ravila Street, Tartu 50411, Estonia

[d]Psychiatry Clinic, Tartu University Hospital, 31 Raja Street, Tartu 50417, Estonia

Supplementary material consists of three separate parts: supplementary figure of behavioral and body weight data, raw metabolic data and summary table of relevant strain-specific metabolites in supplementary tables S1-S9, and quality control data in table S10.

1.Behavioral and body weight data

Supplementary Figure S1.

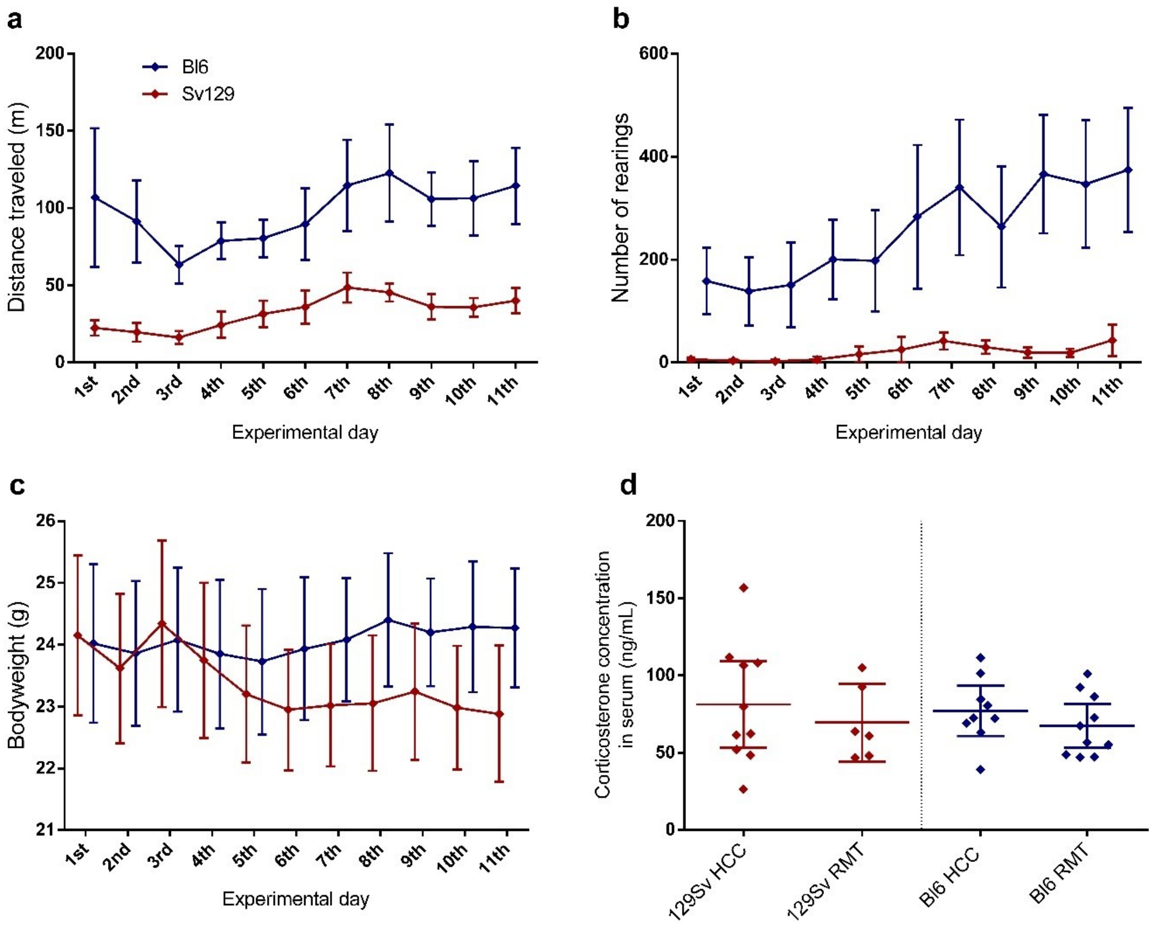

Figure S1. Time course of behavioral effects and body weight changes in 129Sv and Bl6 mice during the repeated testing. By the 11th day of repeated testing the locomotor activity (**a**), rearings (**b**) and body weight (**c**) had stabilized in both strains. Corticosterone levels in home cage controls and repeatedly tested 129Sv and Bl6 mice were measured on 11th day (**d**). HCC – home cage control; RMT – repeated motility tested.

2. Metabolic raw data

Supplementary Table S1.

Comparison of metabolite levels between Bl6 and 129Sv in HCCs (Mann-Whitney U test; p ≤ 0.05). Raw data of marker levels (µM) are presented as median and range. Bonferroni corrected statistically significant results are indicated in bold (p ≤ 0.0003). Eta^2 (eta^2; Z^2/N, where Z - Z – score and N - sample size, N = 22) values of ≥ 0.14 were defined as large effect. Glycerophospholipids include: lysophosphatidylcholine acyls, phosphatidylcholine diacyls, and phosphatidylcholine acyl-alkyls (indicated in italic).

Metabolite	Bl6 (N=12)	129Sv (N=10)	Z-score	*p*-value	Eta^2
	Median (range)	Median (range)			
**Acylcarnitines**					
C0	37.2 (26.2 – 48.8)	41.3 (28.8 – 65.2)	-1.55	0.12	-
C10	0.34 (0.23 – 0.53)	0.38 (0.25 – 0.64)	-0.66	0.51	-
C12	0.070 (0.057 – 0.099)	0.076 (0.055 – 0.086)	-0.36	0.72	-
C14	0.062 (0.044 – 0.117)	0.066 (0.041 – 0.108)	-0.53	0.60	-
C14:1	0.040 (0.025 – 0.063)	0.038 (0.027 – 0.071)	0.43	0.67	-
C14:1-OH	0.016 (0.012 – 0.023)	0.016 (0.010 – 0.022)	0.40	0.69	-
C14:2	0.014 (0.0090 – 0.027)	0.016 (0.014 – 0.019)	-1.91	0.06	-
C16	0.22 (0.15 – 0.34)	0.23 (0.16 – 0.32)	0.03	0.97	-
C16-OH	0.037 (0.025 – 0.060)	0.041 (0.022 – 0.053)	-0.20	0.84	-
C16:1	0.07 (0.048 – 0.111)	0.06 (0.042 – 0.102)	1.15	0.25	-
C16:1-OH	0.018 (0.0080 – 0.031)	0.017 (0.0090 – 0.024)	0.76	0.45	-
C18	0.047 (0.031 – 0.087)	0.053 (0.040 – 0.075)	-1.49	0.13	-
C18:1	0.14 (0.077 – 0.169)	0.1 (0.061 – 0.173)	1.19	0.24	-
C18:2	0.047 (0.031 – 0.078)	0.049 (0.031 – 0.057)	0.30	0.77	-
C2	30.2 (18.2 – 62.1)	35.6 (20.8 – 45.3)	-1.23	0.22	-
C3	0.84 (0.62 – 2.63)	1.04 (0.75 – 1.62)	-1.42	0.16	-

C4	1.03 (0.64 – 2.37)	1.45 (0.80 – 2.17)	-1.95	0.05	0.17
C3-DC (C4-OH)	0.31 (0.12 – 0.76)	0.35 (0.15 – 0.60)	-0.69	0.49	-
**C5**	**0.24 (0.17 – 0.39)**	**0.66 (0.38 – 0.84)**	**-3.86**	**0.0001**	**0.68**
C5-OH (C3-DC-M)	0.120 (0.077 – 0.195)	0.168 (0.103 – 0.242)	-2.21	0.03	0.22
C6 (C4:1-DC)	0.057 (0.027 – 0.082)	0.043 (0.032 – 0.059)	1.19	0.24	-
C5-DC (C6-OH)	0.036 (0.022 – 0.053)	0.037 (0.023 – 0.064)	-0.46	0.64	-
C7-DC	0.019 (0.010 – 0.033)	0.022 (0.011 – 0.027)	-0.82	0.41	-
C8	0.096 (0.071 – 0.133)	0.096 (0.083 – 0.110)	0.20	0.84	-
**Monosaccharides**					
Hexoses	7138 (5367 – 9071)	5423 (3787 – 7761)	2.6	0.009	0.31
**Amino acids and biogenic amines**					
Alanine	395 (273 – 638)	512 (305 – 700)	-1.81	0.07	-
Arginine	90.6 (3.65 – 294)	137 (5 – 480)	-0.76	0.45	-
Asparagine	30.6 (21.2 – 64.0)	45.6 (22.7 – 101)	-1.68	0.09	-
Aspartate	89.7 (25.5 – 195)	73.6 (29.2 – 179)	0.36	0.72	-
Citrulline	35.7 (27.8 – 52.9)	43.5 (35.7 – 53.7)	-1.68	0.09	-
Glutamine	549 (396 – 902)	469 (347 – 656)	1.15	0.25	-
Glutamate	734 (175 – 5870)	1910 (142 – 5240)	-1.29	0.20	-
Glycine	402 (344 – 574)	372 (214 – 539)	1.02	0.31	-
Histidine	66.8 (60.3 – 117)	87.6 (68.3 – 154)	-2.81	0.005	0.36
Isoleucine	101 (70.6 – 149)	124 (88.6 –200)	-2.67	0.008	0.32
Leucine	153 (117 – 347)	201 (158 – 619)	-2.61	0.009	0.31
Lysine	269 (193 – 563)	288 (195 – 678)	0.99	0.32	-
Methionine	47.4 (38.2 – 123)	73.7 (43.6 – 170)	-2.18	0.03	0.22

Ornithine	116 (41.8 – 220)	77.9 (49.9 – 208)	0.30	0.77	-
Phenylalanine	78.0 (64.8 – 192)	90.4 (57.4 – 216)	-1.29	0.20	-
Proline	107 (81.2 – 128)	124 (95.2 – 255)	-1.32	0.10	-
Serine	125 (93.9 – 235)	208 (137 – 370)	-3.2	0.0014	0.47
Threonine	114 (93.1 – 192)	190 (144 – 286)	-2.94	0.003	0.39
Tryptophan	65.8 (46.7 – 101)	81.9 (34.4 – 128)	-1.64	0.10	-
Tyrosine	76.6 (50.7 – 131)	75.9 (50.9 – 172)	0.16	0.87	-
Valine	236 (185 – 378)	302 (228 – 442)	-2.6	0.009	0.31
**Acetyl-ornithine**	**10.6 (8.88 – 14.1)**	**4.53 (3.51 – 5.39)**	**3.92**	**0.00009**	**0.7**
ADMA	0.49 (0.34 – 0.64)	0.54 (0.40 – 0.79)	-0.3	0.77	-
**Alpha-aminoadipic acid**	**10.6 (8.07 – 14.3)**	**5.13 (2.74 – 8.21)**	**3.86**	**0.0001**	**0.68**
Carnosine	8.46 (6.96 – 15.9)	5.24 (2.30 – 8.63)	3.53	0.0004	0.57
Creatinine	13.4 (10.8 – 25.1)	15.2 (11.7 – 20.7)	-1.15	0.25	-
Histamine	2.07 (1.31 – 3.70)	3.08 (1.26 – 4.34)	-1.45	0.15	-
Kynurenine	0.77 (0.56 – 1.20)	0.75 (0.55 – 1.03)	0.36	0.72	-
Met-SO	0.91 (0.58 – 2.03)	2.06 (0.62 – 5.97)	-2.90	0.004	0.38
Putrescine	1.76 (1.02 – 2.78)	1.00 (0.43 – 2.31)	2.08	0.04	0.20
Taurine	649 (521 – 713)	655 (551 – 701)	-0.03	0.97	-
5-HT	14.0 (5.34 – 33.3)	6.76 (3.90 – 23.7)	1.71	0.09	-
Spermidine	14.1 (5.77 – 23.0)	16.3 (4.67 – 27.2)	-0.99	0.32	-
Spermine	4.42 (1.45 – 9.47)	6.88 (3.90 – 23.7)	-1.62	0.11	-
t4-OH-Pro	20.8 (18.8 – 30.3)	15.7 (12.2 – 23.3)	2.6	0.009	0.31
**Glycerophospholipids**					
Lysophosphatidylcholine acyls					
PC(16:0/0:0)	257 (145 – 375)	185 (92.1 – 360)	1.88	0.06	-

**PC(16:1/0:0)**	**8.59 (6.13 – 15.9)**	**3.29 (1.30 – 5.06)**	**3.92**	**0.00009**	**0.7**
PC(17:0/0:0)	3.15 (1.70 – 4.26)	3.82 (2.11 – 6.77)	-1.68	0.09	-
PC(18:0/0:0)	77.6 (37.6 – 108)	73.3 (43.9 – 143)	0.16	0.87	-
PC(18:1/0:0)	62.9 (35.0 – 102)	29.5 (14.7 – 47.4)	3.53	0.0004	0.57
PC(18:2/0:0)	144 (69.0 – 185)	94.9 (39.1 – 144)	2.28	0.02	0.24
**PC(20:3/0:0)**	**11.8 (6.55 –18.1**	**5.69 (2.23 – 8.22)**	**3.66**	**0.0002**	**0.61**
PC(20:4/0:0)	35.2 (16.0 – 47.9)	19.8 (7.11 – 31.1)	3.13	0.002	0.45
PC(24:0/0:0)	1.30 (0.86 – 1.84)	1.69 (1.35 – 2.29)	-3.13	0.002	0.45
PC(26:0/0:0)	1.99 (1.40 – 2.60)	2.35 (1.80 – 3.71)	-1.99	0.05	0.18
PC(26:1/0:0)	0.66 (0.48 – 0.86)	0.72 (0.46 – 1.03)	-1.22	0.22	-
PC(28:0/0:0)	0.80 (0.65 – 1.12)	1.26 (0.90 – 1.78	-3.00	0.003	0.41
PC(28:1/0:0)	0.63 (0.51 – 0.91)	0.72 (0.46 – 1.24)	1.32	0.19	-
Phosphatidylcholine diacyls					
PC aa C24:0	0.52 (0.31 – 0.80)	0.59 (0.44 – 0.94)	-1.68	0.09	-
PC aa C26:0	2.57 (1.98 – 3.83)	3.35 (2.17 – 6.55)	-1.71	0.09	-
PC aa C28:1	0.61 (0.48 – 0.98)	0.80 (0.56 – 1.36)	-1.91	0.06	-
PC aa C30:0	0.84 (0.63 – 1.57)	1.06 (0.79 – 3.35)	-2.67	0.008	0.32
PC aa C30:2	0.093 (0.014 – 0.236)	0.117 (0.022 – 0.229)	-0.69	0.49	-
PC aa C32:0	8.97 (7.14 – 14.8)	12.1 (6.06 – 37.2)	-2.14	0.03	0.21
PC aa C32:1	6.33 (4.41 – 9.05)	4.31 (2.17 – 14.9)	2.67	0.008	0.32
PC aa C32:2	0.80 (0.47 – 1.23)	0.63 (0.45 – 1.26)	1.42	0.16	-
PC aa C32:3	0.103 (0.088 – 0.139)	0.129 (0.086 – 0.155)	-1.62	0.11	-
PC aa C34:1	91.5 (62.7 – 117)	65.2 (56.2 – 102)	2.14	0.03	0.21
PC aa C34:2	237 (170 – 288)	275 (222 – 329)	-2.21	0.03	0.22
**PC aa C34:3**	**9.11 (6.78 – 12.8)**	**4.95 (3.90 – 6.95)**	**3.79**	**0.0001**	**0.65**

PC aa C34:4	0.30 (0.21 – 0.44)	0.23 (0.17 – 0.31)	2.67	0.008	0.32
PC aa C36:0	1.66 (1.26 – 2.26)	2.23 (1.96 – 2.87)	-3.43	0.0006	0.53
PC aa C36:1	11.9 (9.09 – 14.9)	9.75 (6.89 – 14.4)	1.55	0.12	-
PC aa C36:2	117 (79.5 – 142)	165 (118 – 193)	-3.27	0.001	0.49
PC aa C36:3	50.7 (33.5 – 71.6)	49.7 (35.8 – 58.1)	0.73	0.47	-
PC aa C36:4	84.7 (55.4 – 96.6)	83.6 (64.6 – 94.7)	0.00	1.00	-
PC aa C36:5	4.31 (3.13 – 6.86)	3.76 (2.89 – 5.68)	1.78	0.07	-
PC aa C36:6	0.22 (0.15 – 0.32)	0.20 (0.13 – 0.31)	0.79	0.43	-
PC aa C38:0	1.12 (0.69 – 1.56)	1.17 (0.94 – 1.91)	-1.45	0.15	-
PC aa C38:1	0.66 (0.47 – 1.18)	0.74 (0.40 – 1.07)	-0.53	0.60	-
PC aa C38:3	12.7 (9.44 – 17.3)	14.3 (11.0 – 16.9)	-0.89	0.37	-
PC aa C38:4	36.9 (23.3 – 47.0)	39.7 (28.9 – 47.6)	-1.35	0.18	-
PC aa C38:5	17.4 (11.8 – 22.1)	14.8 (10.9 – 19.1)	0.56	0.58	-
PC aa C38:6	70.4 (42.4 – 78.4)	65.1 (44.4 – 83.1)	0.49	0.62	-
PC aa C40:1	0.31 (0.26 – 0.39)	0.34 (0.29 – 0.41)	-1.98	0.05	0.18
PC aa C40:2	0.34 (0.25 – 0.54)	0.48 (0.36 – 0.64)	-2.87	0.004	0.37
PC aa C40:3	0.49 (0.38 – 0.74)	0.59 (0.48 – 0.71)	-1.68	0.09	-
PC aa C40:4	1.66 (1.07 – 1.87)	1.87 (1.35 – 2.32)	-2.18	0.03	0.22
PC aa C40:5	2.11 (1.58 – 2.84)	2.69 (1.85 – 3.53)	-2.74	0.006	0.34
PC aa C40:6	17.5 (10.4 – 21.2)	21.3 (13.8 – 27.1)	-2.24	0.02	0.23
PC aa C42:0	0.106 (0.087 – 0.164)	0.148 (0.125 – 0.189)	-3.30	0.001	0.5
PC aa C42:1	0.116 (0.075 – 0.153)	0.14 (0.12 – 0.16)	-2.64	0.008	0.32

PC aa C42:2	0.23 (0.17 – 0.27)	0.31 (0.23 – 0.36)	-2.94	0.003	0.39
PC aa C42:4	0.17 (0.10 – 0.22)	0.21 (0.15 – 0.29)	-2.34	0.02	0.25
PC aa C42:5	0.21 (0.18 – 0.30)	0.26 (0.18 – 0.30)	-1.29	0.2	-
PC aa C42:6	0.66 (0.48 – 0.83)	0.84 (0.58 – 1.13)	-2.34	0.02	0.25
Phosphatidylcholine acyl-alkyls					
PC ae C30:0	0.26 (0.21 – 0.30)	0.32 (0.25 – 0.50)	-2.80	0.005	0.36
PC ae C30:1	0.38 (0.31 – 0.56)	0.50 (0.35 – 0.96)	-2.14	0.03	0.21
PC ae C30:2	0.108 (0.083 – 0.179)	0.120 (0.091 – 0.167)	0.92	0.36	-
PC ae C32:1	0.50 (0.36 – 0.64)	0.76 (0.56 – 1.08)	-3.40	0.0007	0.53
PC ae C32:2	0.22 (0.17 – 0.33)	0.30 (0.23 – 0.36)	-1.98	0.05	0.18
PC ae C34:0	0.32 (0.25 – 0.44)	0.51 (0.31 – 0.94)	-3.43	0.0006	0.54
PC ae C34:1	2.07 (1.44 – 2.70)	1.88 (1.49 – 2. 69)	0.96	0.34	-
PC ae C34:2	1.84 (1.26 – 2.47)	2.31 (1.97 – 3.19)	-2.77	0.006	0.35
PC ae C34:3	0.53 (0.35 – 0.68)	0.68 (0.54 – 0.84)	-2.77	0.006	0.35
PC ae C36:0	0.38 (0.31 – 0.54)	0.49 (0.41 – 0.61)	-3.03	0.002	0.42
PC ae C36:1	1.88 (1.53 – 2.54)	2.28 (1.85 – 2.84)	-2.70	0.007	0.33
**PC ae C36:2**	**4.67 (3.08 – 6.18)**	**8.73 (6.41 – 10.2)**	**-3.92**	**0.00009**	**0.7**
PC ae C36:3	1.14 (0.79 – 1.53)	1.17 (0.85 – 1.47)	0.10	0.92	-
PC ae C36:4	1.69 (1.15 – 2.12)	1.81 (1.44 – 2.38)	-0.40	0.69	-
PC ae C36:5	0.84 (0.55 – 0.98)	0.96 (0.88 – 1.34)	-2.44	0.01	0.27
PC ae C38:0	2.21 (1.47 – 3.07)	1.70 (1.11 – 3.48)	1.39	0.17	-
PC ae C38:1	0.61 (0.48 – 0.82)	0.87 (0.38 – 1.06)	-2.74	0.006	0.34
**PC ae C38:2**	**3.56 (2.27 – 4.89)**	**8.31 (5.37 – 9.90)**	**-3.92**	**0.00009**	**0.7**
PC ae C38:3	1.10 (0.74 – 1.42)	1.60 (1.21 – 1.83)	-3.40	0.0007	0.53
PC ae C38:4	1.91 (1.26 – 2.26)	2.68 (2.06 – 2.99)	-3.27	0.001	0.49

PC ae C38:5	1.58<br>(1.14 – 2.08)	1.50<br>(1.10 – 1.80)	0.86	0.39	-
PC ae C38:6	1.10<br>(0.80 – 1.36)	1.30<br>(0.94 – 1.64)	-1.55	0.12	-
PC ae C40:1	1.82<br>(1.25 – 2.36)	1.66<br>(1.36 – 2.12)	0.89	0.37	-
PC ae C40:2	0.41<br>(0.31 – 0.60)	0.53<br>(0.46 – 0.62)	-2.93	0.003	0.39
PC ae C40:3	0.42<br>(0.25 – 0.53)	0.54<br>(0.45 – 0.65)	-3.26	0.001	0.48
**PC ae C40:4**	**1.00**<br>**(0.63 – 1.12)**	**1.49**<br>**(1.15 – 1.70)**	**-3.92**	**0.00009**	**0.7**
PC ae C40:5	0.65<br>(0.47 – 0.81)	0.77<br>(0.61 – 0.91)	-2.18	0.03	0.22
**PC ae C40:6**	**1.15**<br>**(0.78 – 1.36)**	**1.75**<br>**(1.30 – 2.08)**	**-3.76**	**0.0002**	**0.64**
PC ae C42:0	0.75<br>(0.64 – 0.94)	0.79<br>(0.58 – 0.90)	0.59	0.55	-
PC ae C42:1	0.39<br>(0.29 – 0.50)	0.46<br>(0.31 – 0.55)	-1.72	0.09	-
PC ae C42:2	0.27<br>(0.18 – 0.37)	0.31<br>(0.23 – 0.36)	-1.55	0.12	-
PC ae C42:3	0.58<br>(0.38 – 0.78)	0.56<br>(0.35 – 0.61)	1.16	0.25	-
PC ae C42:4	0.15<br>(0.10 – 0.22)	0.18<br>(0.13 – 0.21)	-2.41	0.02	0.26
PC ae C42:5	0.50<br>(0.39 – 0.57	0.57<br>(0.50 – 0.65)	-2.93	0.003	0.39
PC ae C44:3	0.086<br>(0.065 – 0.124)	0.126<br>(0.079 – 0.145)	-2.61	0.009	0.31
PC ae C44:4	0.114<br>(0.088 – 0.144)	0.116<br>(0.101 – 0.162)	-0.89	0.37	-
PC ae C44:5	0.125<br>(0.094 – 0.165)	0.120<br>(0.099 – 0.154)	0.56	0.58	-
PC ae C44:6	0.094<br>(0.075 – 0.119)	0.102<br>(0.091 – 0.126)	-1.78	0.07	-
**Sphingolipids**					
**SM (OH) C14:1**	**0.25**<br>**(0.14 – 0.36)**	**0.51**<br>**(0.37 – 0.66)**	**-3.92**	**0.00009**	**0.7**
SM (OH) C16:1	0.082<br>(0.061– 1.020)	0.103<br>(0.068 – 0.159)	-2.43	0.01	0.27
**SM (OH) C22:1**	**0.54**<br>**(0.40 – 0.67)**	**0.85**<br>**(0.67 – 1.02)**	**-3.92**	**0.00009**	**0.7**
SM (OH) C22:2	0.33<br>(0.27 – 0.43)	0.48<br>(0.39 – 0.64)	-3.46	0.0005	0.54
SM (OH) C24:1	0.075<br>(0.046 – 0.180)	0.133<br>(0.096 – 0.255)	-2.28	0.02	0.24
SM C16:0	4.83<br>(3.45 – 6.72)	6.13<br>(4.79 – 9.21)	-2.34	0.02	0.25

SM C16:1	1.02<br>(0.67– 1.37)	1.39<br>(1.09 – 2.04)	-2.93	0.003	0.39
SM C18:0	0.48<br>(0.37 – 0.72)	0.76<br>(0.38 – 1.40)	-2.41	0.02	0.26
SM C18:1	0.16<br>(0.12 – 0.23)	0.19<br>(0.14 – 0.29)	-1.85	0.06	-
SM C20:2	0.002<br>(0.000 – 0.032)	0.013<br>(0.000 – 0.025)	-1.40	0.16	-
SM C22:3	0.000<br>(0.000 – 0.061)	0.000<br>(0.000 – 0.055)	-0.10	0.92	-
**SM C24:0**	**2.43<br>(1.88– 2.60)**	**3.23<br>(2.77 – 4.44)**	**-3.92**	**0.00009**	**0.7**
SM C24:1	3.80<br>(2.85 – 5.00)	3.97<br>(2.94 – 4.87)	-0.89	0.37	-
SM C26:0	0.008<br>(0.000 – 0.026)	0.025<br>(0.000 – 0.049)	-1.78	0.08	-
SM C26:1	0.012<br>(0.000 – 0.043)	0.030<br>(0.000 – 0.067)	-1.03	0.30	-

Supplementary Table S2.

Comparison of amino acid levels and Fisher ratio for Bl6 and 129Sv in HCC (Mann-Whitney U test; $p \le 0.05$). Raw data of marker levels (µM) are presented as median and range. Bonferroni corrected statistically significant results are indicated in bold ($p \le 0.0003$). Eta^2 (eta^2; Z^2/N, where Z - Z – score and N - sample size, N = 22) values of ≥ 0.14 were defined as large effect. *Fisher ratio – ratio between the branched-chain and aromatic amino acids.

Metabolite	Bl6 (N=12)	129Sv (N=10)	Z-score	*p*-value	Eta^2
	Median (range)	Median (range)			
Aromatic amino acids	233 (162 – 363)	251 (143 – 505)	-1.35	0.18	-
Branched-chain amino acids	488 (388 – 874)	614 (475 – 1261)	-2.60	0.009	0.31
*Fisher ratio	2.14 (1.79 – 2.50)	2.51 (2.09 – 3.33)	-2.94	0.003	0.39
Glycolysis-related amino acids	883 (728 – 1408)	1078 (695 – 1499)	-1.42	0.16	-
Non-essential amino acids	3208 (1930 – 8084)	4141 (1674 – 7557)	-1.09	0.27	-
Essential amino acids	1064 (859 – 2001)	1349 (959 – 2497)	-2.34	0.02	0.25

Supplementary Table S3.

Metabolite ratios elevated in Bl6 mice HCCs (Mann-Whitney U test; $p \le 0.05$). Raw data of marker levels (µM) are presented as median and range. Bonferroni corrected statistically significant results are indicated in bold ($p \le 0.0003$). Eta^2 (eta^2; Z^2/N, where Z - Z – score and N - sample size, N = 22) values of ≥ 0.14 were defined as large effect.

Metabolite	Bl6 (N=12)	129Sv (N=10)	Z-score	*p*-value	Eta^2
	Median (range)	Median (range)			
C3 / C4	0.93 (0.73 – 154)	0.76 (0.67 – 1.01)	1.95	0.05	0.17
**C4 / C5**	**3.71 (3.51 – 6.67)**	**2.13 (1.75 – 3.10)**	**3.92**	**0.00009**	**0.7**
Glycine / histidine	5.70 (4.35 – 7.70)	3.76 (2.64 – 5.04)	3.46	0.005	0.54
**Glycine / PC ae 38:2**	**117 (78.4 – 210)**	**48.2 (25.1 – 70.6)**	**3.92**	**0.00009**	**0.7**
**Glycine / serine**	**3.29 (2.36 –3.90)**	**1.82 (1.17 – 2.39)**	**3.86**	**0.0001**	**0.68**
Tyrosine / phenylalanine	0.94 (0.84 – 1.09)	0.75 (0.72 – 0.87)	2.35	0.02	0.25

Supplementary Table S4.

Metabolite ratios elevated in 129Sv mice HCCs (Mann-Whitney U test; p ≤ 0.05). Raw data of marker levels (µM) are presented as median and range. Raw data of marker levels (µM) are presented as median and range. Bonferroni corrected statistically significant results are indicated in bold (p ≤ 0.0003). Eta^2 (eta^2; Z^2/N, where Z - Z – score and N - sample size, N = 22) values of ≥ 0.14 were defined as large effect.

Metabolite	Bl6 (N=12)	129Sv (N=10)	Z-score	*p*-value	Eta^2
	Median (range)	Median (range)			
**C5 / C0**	**0.0066 (0.0039 – 0.0098)**	**0.015 (0.0099 - 0.023)**	**-3.89**	**0.0001**	**0.68**
C14 / C16:1	0.84 (0.71 – 1.54)	1.08 (0.85 – 1.41)	-2.41	0.02	0.26
C18 / C18:1	0.39 (0.25 – 0.58)	0.50 (0.38 – 0.79)	-2.27	0.02	0.23
**PC(16:0/0:0) / PC(16:1/0:0)**	**27.4 (20.7 – 34.7)**	**60.7 (42.9 –85.3)**	**-3.92**	**0.00009**	**0.7**
PC aa C40:5 / PC aa C42:5	9.70 (8.08 – 11.3)	10.9 (9.31 – 12.8)	-2.41	0.02	0.26
**Spermidine / Putrescine**	**7.99 (2.86 – 11.4)**	**12.3 (10.8 – 33.5)**	**-3.86**	**0.0001**	**0.68**

Supplementary Table S5.

Comparison of metabolite levels between Bl6 and 129Sv (Mann-Whitney U test; p ≤ 0.05) in RMT. Raw data of marker levels (µM) are presented as median and range. Bonferroni corrected statistically significant results are indicated in bold (p ≤ 0.0003). Eta^2 (eta^2; Z^2/N, where Z - Z – score and N - sample size, N = 22) values of ≥ 0.14 were defined as large effect. Glycerophospholipids include: lysophosphatidylcholine acyls, phosphatidylcholine diacyls, and phosphatidylcholine acyl-alkyls (indicated in italic).

Metabolite	Bl6 (N=12)	129Sv (N=11)	Z-score	*p*-value	Eta^2
	Median (range)	Median (range)			
**Acylcarnitines**					
C0	43.6 (21.6 – 62.5)	25.9 (15.5 – 53.7)	2.06	0.04	0.18
C10	0.17 (0.000 – 0.194)	0.000 (0.000 – 0.161)	2.95	0.003	0.38
C12	0.092 (0.000 – 0.142)	0.094 (0.000 – 0.122)	0.09	0.93	-
C14	0.091 (0.060 – 0.119)	0.081 (0.067 – 0.101)	0.43	0.67	-
C14:1	0.063 (0.039 – 0.082)	0.057 (0.036 – 0.073)	1.29	0.2	-
C14:1-OH	0.016 (0.000 – 0.020)	0.013 (0.000 – 0.019)	1.63	0.1	-
C14:2	0.021 (0.000 – 0.026)	0.015 (0.000 – 0.021)	2	0.05	0.17
C16	0.34 (0.18 – 0.40)	0.27 (0.21 – 0.34)	1.26	0.21	-
C16-OH	0.026 (0.000 – 0.037)	0.022 (0.000 – 0.026)	1.85	0.07	-
C16:1	0.12 (0.063 – 0.144)	0.08 (0.059 – 0.098)	2.95	0.003	0.38
C16:1-OH	0.019 (0.000 – 0.027)	0.015 (0.000 – 0.019)	2.37	0.02	0.24
C18	0.061 (0.034 – 0.072)	0.053 (0.049 – 0.077)	0.31	0.76	-
C18:1	0.26 (0.15 – 0.36)	0.15 (0.14 – 0.20)	3.26	0.001	0.46
C18:2	0.088 (0.051 – 0.121)	0.063 (0.055 – 0.091)	1.78	0.07	-
C2	34.2 (28.3 – 45.6)	32.1 (28.5 – 38.8)	0.89	0.37	-
C3	1.07 (0.58 – 1.45)	0.71 (0.35 – 1.58)	1.51	0.13	-
C4	0.89 (0.61 – 1.22)	1.29 (0.67 – 2.19)	-3.05	0.002	0.41

C3-DC (C4-OH)	0.35<br>(0.21 – 0.57)	0.30<br>(0.17 – 0.40)	1.08	0.28	-
**C5**	**0.22<br>(0.18 – 0.28)**	**0.4<br>(0.23 –0.63)**	**-3.79**	**0.0002**	**0.63**
C5-OH (C3-DC-M)	0.146<br>(0.000 – 0.182)	0.139<br>(0.000 – 0.223)	-0.68	0.5	-
C6 (C4:1-DC)	0.064<br>(0.044 – 0.099)	0.058<br>(0.037 – 0.085)	1.45	0.15	-
C5-DC (C6-OH)	0.036<br>(0.000 – 0.044	0.034<br>(0.000 – 0.042)	0.77	0.44	-
C7-DC	0.014<br>(0.000 – 0.074)	0.000<br>(0.000 –0.047)	0.83	0.41	-
C8	0.117<br>(0.000 – 0.159)	0.000<br>(0.000 –0.141)	2.28	0.02	0.23
**Monosaccharides**					
Hexoses	8777<br>(4935 – 11103)	5810<br>(4405 – 8005)	3.35	0.0008	0.49
**Amino acids and biogenic amines**					
Alanine	526<br>(344 – 991)	410<br>(296 – 677)	1.32	0.19	-
Arginine	70.6<br>(3.67 – 146)	31.7<br>(2.79 – 102)	1.32	0.19	-
Asparagine	20.5<br>(16.9 – 41.5)	25.9<br>(19.6 – 38.8)	-1.72	0.09	-
Aspartate	83.2<br>(19.3 – 266)	86.9<br>(15.2 – 182)	0.68	0.5	-
Citrulline	52.1<br>(40.0 – 112)	49.5<br>(28.9 – 111)	0.52	0.6	-
Glutamine	853<br>(509 – 1120)	727<br>(605 – 1090)	1.57	0.12	-
Glutamate	1340<br>(283 – 3760)	519<br>(77.9 – 1900)	2.03	0.04	0.18
Glycine	544<br>(184 – 772)	322<br>(180 – 467)	2.8	0.005	0.34
Histidine	70.5<br>(44.4 – 81.9)	70.1<br>(55.7 – 120)	-0.22	0.83	-
Isoleucine	83.7<br>(60.6 – 108)	97.3<br>(79.8 – 129)	-2.49	0.01	0.27
Leucine	122<br>(92.8 – 159)	137<br>(111 – 197)	-2	0.046	0.17
Lysine	267<br>(159 – 397)	240<br>(140 – 399)	1.51	0.13	-
Methionine	38.6<br>(30.0 – 56.7)	41.9<br>(28.9 – 73.6)	-1.02	0.31	-
Ornithine	77.3<br>(24.1 – 146)	77.5<br>(20.2 – 188)	0.34	0.74	-

Phenylalanine	82.1 (54.5 – 95.5)	74.5 (52.1 – 94.5)	1.45	0.15	**-**
Proline	85.5 (54.3 – 138)	71.8 (45.7 – 116)	2.43	0.02	0.26
Serine	149 (89.2 – 248)	145 (90.2 – 238)	0.43	0.67	-
Threonine	134 (89.8 – 169)	134 (101 – 233)	-0.31	0.76	-
Tryptophan	73.4 (56.3 – 114)	68.7 (52.2 – 95.3)	1.20	0.23	-
Tyrosine	56.8 (41.0 – 74.2)	46.6 (27.4 – 56.5)	2.37	0.02	0.24
Valine	159 (137 – 208)	192 (146 – 297)	-2.4	0.02	0.25
**Acetyl-ornithine**	**15.9 (10.6 – 19.1)**	**7.25 (5.40 – 12.1)**	**3.91**	**0.00009**	**0.67**
ADMA	0.356 (0.000 – 0.752)	0.283 (0.109 – 0.525)	1.08	0.28	-
**Alpha-aminoadipic acid**	**10.95 (7.420 – 17.200)**	**0.000 (0.000 – 9.490)**	**3.85**	**0.0001**	**0.65**
**Carnosine**	**15.5 (3.20 – 21.2)**	**2.79 (1.17 – 7.34)**	**3.72**	**0.0002**	**0.6**
Creatinine	16.6 (7.80 – 25.6)	10.2 (5.11 – 19.5)	2.40	0.02	0.25
Histamine	2.41 (0.31 – 4.03)	2.60 (1.12 – 7.01)	-0.77	0.44	-
Kynurenine	1.4 (0.93 – 1.56)	1.43 (1.13 – 1.90)	-0.77	0.44	-
Met-SO	0.000 (0.000 – 1.610)	0.000 (0.000 – 2.050)	0.28	0.78	-
Putrescine	1.145 (0.000 – 1.840)	0.594 (0.307 – 1.150)	2.15	0.03	0.2
Taurine	884 (723 – 1040)	819 (451 – 1000)	1.11	0.27	-
5-HT	11.5 (6.28 – 25.9)	5.91 (2.50 – 18.8)	2.18	0.03	0.21
Spermidine	15.1 (4.62 – 40.6)	9.15 (4.61 – 18.1)	1.85	0.07	-
Spermine	5.53 (2.35 – 14.8)	4.73 (1.38 – 10.4)	0.52	0.6	-
t4-OH-Pro	23.6 (7.54 – 38.5)	13.3 (5.80 – 21.3)	2.83	0.005	0.35
**Glycerophospholipids**					
**Lysophosphatidylcholine acyls**					
PC(16:0/0:0)	312 (158 – 376)	292 (179 – 444)	0.31	0.76	-

**PC(16:1/0:0)**	**12.75 (6.31 – 17.8)**	**5.87 (3.50 – 8.26)**	**3.79**	**0.0002**	**0.62**
PC(17:0/0:0)	3.52 (1.89 – 4.64)	4.58 (3.24 – 6.56)	-2.28	0.02	0.23
PC(18:0/0:0)	98.4 (47.9 – 114)	105 (62.5 – 173)	-1.02	0.31	-
PC(18:1/0:0)	81.1 (39.5 – 110)	59.8 (34.8 – 86.1)	2.18	0.03	0.21
PC(18:2/0:0)	160 (98.7 – 198)	133 (88.0 – 181)	1.11	0.27	-
PC(20:3/0:0)	12.5 (6.40 – 17.7)	8.94 (4.98 – 13.8)	2.65	0.008	0.31
PC(20:4/0:0)	49.4 (20.0 – 59.2)	29.3 (20.2 – 44.0)	3.05	0.002	0.41
PC(24:0/0:0)	0.92 (0.44 – 1.10)	0.95 (0.56 – 1.47)	-0.77	0.44	-
PC(26:0/0:0)	1.11 (0.43 – 1.46)	0.92 (0.36 – 1.68)	0.89	0.37	-
PC(26:1/0:0)	0.36 (0.16 – 0.57)	0.30 (0.15 – 0.69)	0.80	0.42	-
PC(28:0/0:0)	0.54 (0.27 – 0.73)	0.49 (0.18 – 0.91)	0.83	0.41	-
PC(28:1/0:0)	0.47 (0.19 – 0.59)	0.29 (0.11 – 0.73)	1.45	0.15	-
**Phosphatidylcholine diacyls**					
PC aa C24:0	0.42 (0.16 – 0.69)	0.29 (0.18 – 0.52)	1.45	0.15	-
PC aa C26:0	1.770 (0.000 – 2.680)	1.460 (0.000 – 2.410)	0.55	0.58	-
PC aa C28:1	0.48 (0.19 – 0.69)	0.41 (0.20 – 0.66)	0.55	0.58	-
PC aa C30:0	1.00 (0.53 – 2.04)	0.86 (0.53 – 1.41)	1.88	0.06	-
PC aa C30:2	0.078 (0.028 – 0.166)	0.070 (0.019 – 0.159)	0.40	0.69	-
PC aa C32:0	12.0 (6.95 – 22.1)	10.8 (8.34 – 15.9)	0.58	0.56	-
PC aa C32:1	8.05 (4.14 – 11.4)	4.58 (2.87 – 6.29)	3.35	0.0008	0.49
PC aa C32:2	1.20 (0.67 – 1.37)	0.74 (0.44 – 0.82)	3.11	0.002	0.42
PC aa C32:3	0.119 (0.072 – 0.188)	0.090 (0.064 – 0.177)	1.63	0.10	-
PC aa C34:1	98.6 (67.9 – 136)	105 (70.1 – 157)	-0.74	0.46	-
PC aa C34:2	228 (172 – 299)	263 (189 – 356)	-1.54	0.12	-
PC aa C34:3	10.9 (6.75 – 14.5)	5.80 (4.46 – 8.13)	3.54	0.0004	0.55

PC aa C34:4	0.48 (0.24 – 0.53)	0.23 (0.17 – 0.31)	3.42	0.0006	0.51
PC aa C36:0	1.86 (1.25 – 2.61)	1.78 (1.41 – 2.49)	-0.06	0.95	-
PC aa C36:1	16.6 (8.34 – 21.5)	15.5 (10.8 – 21.9)	0.06	0.95	-
PC aa C36:2	125 (86.9 – 147)	175 (111 – 214)	-3.20	0.001	0.45
PC aa C36:3	58.6 (35.1 – 73.6)	58.6 (39.1 – 78.1)	-0.03	0.98	-
PC aa C36:4	102 (50.1 – 124)	88.9 (65.4 – 116)	0.74	0.46	-
PC aa C36:5	5.29 (3.61 – 6.97)	3.64 (2.31 – 5.08)	2.83	0.005	0.35
PC aa C36:6	0.31 (0.18 – 0.41)	0.20 (0.13 – 0.32)	2.68	0.007	0.31
PC aa C38:0	1.05 (0.52 – 1.53)	0.89 (0.68 – 1.34)	0.83	0.41	-
PC aa C38:1	0.94 (0.42 – 1.19)	0.65 (0.54 – 1.11)	1.54	0.12	-
PC aa C38:3	15.5 (9.06 – 20.2)	17.5 (11.0 – 26.7)	-1.35	0.18	-
PC aa C38:4	53.0 (24.2 – 62.4)	44.6 (35.9 – 74.0)	0.34	0.74	-
PC aa C38:5	25.2 (11.8 – 33.3)	20.1 (15.7 – 30.3)	0.34	0.74	-
PC aa C38:6	67.9 (36.1 – 80.6)	68.2 (49.2 – 99.2)	-0.34	0.74	-
PC aa C40:2	0.44 (0.19 – 0.56)	0.44 (0.31 – 0.60)	-0.58	0.56	-
PC aa C40:3	0.59 (0.34 – 0.82)	0.64 (0.40 – 0.83)	-0.18	0.85	-
PC aa C40:4	1.98 (1.05 – 2.50)	1.97 (1.51 – 2.82)	-0.62	0.54	-
PC aa C40:5	3.42 (2.04 – 4.09)	4.23 (3.18 – 6.22)	-2.55	0.01	0.28
PC aa C40:6	18.7 (11.4 – 23.1)	24.1 (15.6 – 37.2)	-2.49	0.01	0.27
PC aa C42:0	0.156 (0.065 – 0.215)	0.171 (0.119 – 0.202)	-1.02	0.31	-
PC aa C42:1	0.152 (0.084 – 0.188)	0.147 (0.128 – 0.193)	-0.03	0.98	-
PC aa C42:2	0.24 (0.14 – 0.30)	0.23 (0.18 – 0.35)	-0.92	0.36	-
PC aa C42:4	0.200 (0.095 – 0.279)	0.222 (0.164 – 0.270)	-0.95	0.34	-
PC aa C42:5	0.30 (0.13 – 0.39)	0.29 (0.18 – 0.38)	0.09	0.93	-
PC aa C42:6	0.73 (0.45 – 0.96)	0.78 (0.61 – 1.07)	-0.77	0.44	-

Phosphatidylcholine acyl-alkyls					
PC ae C30:0	0.205<br>(0.000 – 0.277)	0.180<br>(0.000 – 0.279)	1.08	0.28	-
PC ae C30:1	0.270<br>(0.109 – 0.491)	0.231<br>(0.071 – 0.438)	0.58	0.56	-
PC ae C30:2	0.101<br>(0.042 – 0.129)	0.074<br>(0.051 – 0.135)	1.38	0.17	-
PC ae C32:1	0.57<br>(0.29 – 0.66)	0.49<br>(0.37 – 0.73)	0.03	0.98	-
PC ae C32:2	0.23<br>(0.11 – 0.30)	0.23<br>(0.14 – 0.29)	0.52	0.60	-
PC ae C34:0	0.37<br>(0.23 – 0.48)	0.40<br>(0.31 – 0.49)	-1.17	0.24	-
PC ae C34:1	2.43<br>(1.53 – 3.34)	2.16<br>(1.61 – 2.96)	1.11	0.27	-
PC ae C34:2	1.99<br>(1.28 – 2.56)	2.24<br>(1.53 – 3.10)	-1.11	0.28	-
PC ae C34:3	0.53<br>(0.39 – 0.73)	0.71<br>(0.48 – 1.00)	-2.18	0.03	0.21
PC ae C36:0	0.51<br>(0.22 – 0.87)	0.41<br>(0.27 – 0.51)	1.57	0.12	-
PC ae C36:1	2.47<br>(1.29 – 3.73)	2.45<br>(1.76 – 3.62)	-0.03	0.98	-
PC ae C36:2	5.32<br>(3.36 – 6.99)	7.96<br>(5.33 – 10.1)	-3.05	0.002	0.4
PC ae C36:3	1.19<br>(0.66 – 1.77)	1.08<br>(0.85 – 1.43)	1.20	0.23	-
PC ae C36:4	2.31<br>(1.07 – 2.80)	1.72<br>(1.25 – 2.17)	2.18	0.03	0.21
PC ae C36:5	1.11<br>(0.68 – 1.43)	0.99<br>(0.78 – 1.60)	0.28	0.78	-
PC ae C38:0	2.18<br>(1.05 – 3.11)	1.43<br>(0.98 – 1.99)	2.43	0.02	0.26
PC ae C38:1	0.90<br>(0.40 – 1.16)	0.80<br>(0.52 – 1.04)	0.55	0.58	-
PC ae C38:2	4.09<br>(2.42 – 5.35)	7.27<br>(4.46 – 8.52)	-3.51	0.0005	0.54
PC ae C38:3	1.33<br>(0.74 – 1.79)	1.62<br>(1.17 – 2.10)	-1.42	0.16	-
PC ae C38:4	2.49<br>(1.18 – 2.94)	2.55<br>(1.84 – 3.24)	-0.98	0.33	-
PC ae C38:5	2.10<br>(1.09 – 2.87)	1.66<br>(1.19 – 2.11)	2.25	0.03	0.23
PC ae C38:6	1.21<br>(0.72 – 1.46)	1.18<br>(0.82 – 1.42)	0.09	0.93	-
PC ae C40:1	2.07<br>(1.28 – 2.63)	1.49<br>(1.30 – 1.96)	2.03	0.04	0.18
PC ae C40:2	0.55<br>(0.23 – 0.74)	0.60<br>(0.36 – 0.75)	-1.26	0.21	-

PC ae C40:3	0.56 (0.28 – 0.68)	0.61 (0.41 – 0.82)	-1.51	0.13	-
PC ae C40:4	1.39 (0.61 – 1.71)	1.52 (1.15 – 1.88)	-1.60	0.11	-
PC ae C40:5	0.87 (0.44 – 1.14)	0.78 (0.63 – 1.17)	-0.34	0.74	-
PC ae C40:6	1.32 (0.68 – 1.64)	1.61 (1.14 – 2.11)	-2.77	0.006	0.33
PC ae C42:1	0.49 (0.22 – 0.61)	0.46 (0.41 – 0.68)	0.09	0.93	-
PC ae C42:2	0.40 (0.18 – 0.50)	0.32 (0.24 – 0.48)	0.83	0.41	-
PC ae C42:3	0.83 (0.38 – 1.11)	0.55 (0.46 – 0.89)	1.94	0.05	0.16
PC ae C42:4	0.201 (0.081 – 0.299)	0.207 (0.106 – 0.262)	-0.09	0.93	-
PC ae C44:3	0.107 (0.045 – 0.125)	0.107 (0.065 – 0.151)	0.06	0.95	-
PC ae C44:4	0.120 (0.092 – 0.153)	0.120 (0.090 – 0.155)	-0.06	0.95	-
PC ae C44:5	0.158 (0.000 – 0.211)	0.124 (0.000 – 0.186)	0.95	0.34	-
PC ae C44:6	0.131 (0.065 – 0.170)	0.114 (0.070 – 0.159)	0.95	0.34	-
**Sphingolipids**					
SM (OH) C14:1	0.52 (0.33 – 0.56)	0.74 (0.44 – 1.14)	-3.05	0.002	0.41
SM (OH) C22:1	1.00 (0.69 – 1.17)	1.44 (0.91 – 1.91)	-3.02	0.003	0.4
SM (OH) C22:2	0.64 (0.44 – 0.96)	1.00 (0.66 – 1.27)	-3.02	0.003	0.4
SM (OH) C24:1	0.138 (0.080 – 0.207)	0.190 (0.131 – 0.237)	-2.71	0.007	0.32
SM C16:0	8.25 (6.52 – 10.1)	9.67 (6.67 – 14.0)	-1.88	0.06	-
SM C16:1	1.64 (1.08 – 2.08)	1.79 (1.32 – 2.74)	-1.51	0.13	-
SM C18:0	0.96 (0.64 – 1.28)	0.92 (0.69 – 1.38)	0.22	0.83	-
SM C18:1	0.27 (0.18 – 0.34)	0.28 (0.22 – 0.42)	-0.89	0.37	-
SM C20:2	0.000 (0.000 – 0.024)	0.013 (0.000 – 0.034)	-1.48	0.14	-
SM C24:0	3.44 (2.53 – 4.77)	4.42 (2.96 – 6.02)	-2.31	0.02	0.23
SM C24:1	6.31 (3.76 – 8.35)	9.24 (6.66 – 13.8)	-3.29	0.001	0.47
SM C26:0	0.023 (0.000 – 0.049)	0.021 (0.000 – 0.055)	0.31	0.76	-

SM C26:1	0.016<br>(0.000 – 0.080)	0.036<br>(0.000 – 0.069)	-1.08	0.28	-

Supplementary Table S6.

Comparison of amino acid levels and Fisher ratio in RMT batch for Bl6 and 129Sv (Mann-Whitney U test; p ≤ 0.05). Raw data of marker levels (µM) are presented as median and range. Bonferroni corrected statistically significant results are indicated in bold (p ≤ 0.0003). Eta^2 (eta^2; Z^2/N, where Z - Z – score and N - sample size, N = 22) values of ≥ 0.14 were defined as large effect. *Fisher ratio – ratio between the branched-chain and aromatic amino acids (BCAA/AAA).

Metabolite	Bl6 (N=12)	129Sv (N=11)	Z-score	*p*-value	Eta^2
	Median (range)	Median (range)			
Aromatic amino acids	218 (153 – 259)	184 (152 – 246)	2.16	0.03	0.20
Branched-chain amino acids	373 (308 – 467)	428 (349 – 611)	-2.49	0.01	0.27
***Fisher ratio**	**1.77 (1.45 – 2.10)**	**2.35 (2.11 – 2.65)**	**-4.03**	**0.00006**	**0.71**
Glycolysis-related amino acids	1210 (620 – 1815)	853 (570 – 1372)	2.12	0.03	0.20
Non-essential amino acids	3974 (2159 – 6864)	2636 (1682 – 5204)	2.25	0.02	0.22
Essential amino acids	1025 (714 – 1178)	960 (815 – 1427)	0.15	0.87	-

Supplementary Table S7.

Metabolite ratios elevated in Bl6 (Mann-Whitney U test; p ≤ 0.05) RMT batch. Raw data of marker levels (µM) are presented as median and range. Bonferroni corrected statistically significant results are indicated in bold (p ≤ 0.0003). Eta^2 (eta^2; Z^2/N, where Z - Z – score and N - sample size, N = 22) values of ≥ 0.14 were defined as large effect.

Metabolite	Bl6 (N=12)	129Sv (N=11)	Z-score	*p*-value	Eta^2
	Median (range)	Median (range)			
**C3 / C4**	**1.21 (0.95 – 1.67)**	**0.60 (0.35 – 1.07)**	**3.76**	**0.0002**	**0.61**
C4 / C5	3.95 (2.81 – 5.81)	2.92 (1.85 – 3.95)	2.22	0.03	0.21
Glycine / glutamine	0.59 (0.35 – 0.81)	0.42 (0.30 – 0.51)	2.86	0.004	0.36
Glycine / histidine	7.62 (3.66 – 9.59)	4.25 (2.77 – 5.22)	3.05	0.002	0.40
**Glycine / PC ae 38:2**	**123 (67.1 – 215)**	**48.5 (30.9 – 74.0)**	**3.94**	**0.00008**	**0.67**
Glycine / serine	3.72 (1.70 – 4.39)	2.06 (1.66 – 3.22)	2.65	0.008	0.31
Tyrosine / phenylalanine	0.72 (0.66 – 0.79)	0.62 (0.59 –0.72)	1.67	0.09	-

Supplementary Table S8.

Metabolite ratios elevated in 129Sv (Mann-Whitney U test; p ≤ 0.05) RMT batch. Raw data of marker levels (µM) are presented as median and range. Bonferroni corrected statistically significant results are indicated in bold (p ≤ 0.0003). Eta^2 (eta^2; Z^2/N, where Z - Z – score and N - sample size, N = 22) values of ≥ 0.14 were defined as large effect.

Metabolite	Bl6 (N=12)	129Sv (N=11)	Z-score	*p*-value	Eta^2
	Median (range)	Median (range)			
C2+C3 / C0	0.79 (0.61 – 1.35)	1.23 (0.73 – 1.96)	-2.03	0.04	0.18
C2 / C0	0.77 (0.59 – 1.31)	1.20 (0.70 – 1.69)	-2.03	0.04	0.18
**C4 / C0**	**0.019 (0.013 – 0.041)**	**0.050 (0.022 – 0.075)**	**-3.60**	**0.0003**	**0.56**
**C5 / C0**	**0.0051 (0.0040 – 0.0081)**	**0.014 (0.0096 – 0.023)**	**-4.06**	**0.00005**	**0.72**
**C14 / C16:1**	**0.80 (0.60 – 0.97)**	**1.12 (0.92 – 1.37)**	**-3.66**	**0.0003**	**0.58**
**C16 / C16:1**	**2.87 (2.19 – 3.14)**	**3.44 (2.99 – 4.49)**	**-3.60**	**0.0003**	**0.56**
**C18 / C18:1**	**0.23 (0.17 – 0.34)**	**0.36 (0.33 – 0.39)**	**-3.97**	**0.00007**	**0.69**
**PC(16:0/0:0) / PC(16:1/0:0)**	**23.5 (17.6 – 32.4)**	**51.9 (42.9 –58.5)**	**-4.03**	**0.00006**	**0.71**
PC aa C40:5 / PC aa C42:5	11.7 (9.86 – 15.2)	15.6 (10.6 – 23.9)	-2.62	0.009	0.30
Spermidine / Putrescine	10.1 (0.000 – 21.7)	15.6 (9.83 – 22.1)	-2.52	0.01	0.28

Supplementary Table S9.

Table S9. Relevant strain-specific markers and body weight for both strains (129Sv and Bl6) in both batches (HCC and RMT). Median values of raw data marker levels (µM) with corresponding Z-scores that survived Bonferroni correction are indicated in black ($p \leq 0.0003$). HCC – home cage control; RMT – repeated motility tested.

	HCC			RMT		
**Metabolites**	Bl6	129Sv	Z	Bl6	129Sv	Z
Dominant in Bl6						
acetylornithine	10.6	4.53	3.92	15.9	7.25	3.91
alpha-aminodipic acid	10.6	5.13	3.86	10.95	0.00	3.85
carnosine	8.46	5.24	3.53	15.5	2.79	3.72
PC(16:1/0:0)	8.59	3.29	3.92	12.75	5.87	3.79
PC(20:3/0:0)	11.8	5.69	3.66	12.5	8.94	2.65
PC aa C34:3	9.11	4.95	3.79	10.9	5.80	3.54
Dominant in 129Sv						
C5	0.24	0.66	-3.86	0.22	0.40	-3.79
PC ae C36:2	4.67	8.73	-3.92	5.32	7.96	-3.05
PC ae C38:2	3.56	8.31	-3.92	4.09	7.27	-3.51
PC ae C40:4	1.00	1.49	-3.92	1.39	1.52	-1.60
PC ae C40:6	1.15	1.75	-3.76	1.32	1.61	-2.77
SM (OH) C14:1	0.25	0.51	-3.92	0.52	0.74	-3.05
SM (OH) C22:1	0.54	0.85	-3.92	1.00	1.44	-3.02
SM C24:0	2.43	3.23	-3.92	3.44	4.42	-2.31
**Ratios**						
Dominant in Bl6						
C3/C4	0.93	0.76	1.95	1.21	0.60	3.76
C4/C5	3.71	2.13	3.92	3.95	2.92	2.22
glycine/PC ae 38:2	117	48.2	3.92	123	48.5	3.94
glycine / serine	3.29	1.82	3.86	3.72	2.06	2.65
Dominant in 129Sv						
C4/C0	0.027	0.034	-1.75	0.019	0.050	-3.60
C5/C0	0.007	0.015	-3.89	0.005	0.014	-4.06
C14 / C16:1	0.84	1.08	-2.41	0.80	1.12	-3.66
C16 / C16:1	2.95	3.54	-1.48	2.87	3.44	-3.60
C18 / C18:1	0.39	0.50	-2.27	0.23	0.36	-3.97
PC(16:0/0:0) / PC(16:1/0:0)	27.4	60.7	-3.92	23.5	51.9	-4.03
spermidine / putrescine	7.99	12.3	-3.86	10.1	15.6	-2.52

3. Quality control

Metabolite quantitation

Biocrates AbsoluteIDQ p180 kit (Biocrates Life Sciences AG, Innsbruck, Austria) enables the measurement of 160 endogenous metabolites and 45 metabolite ratios using a combination of flow injection analysis and liquid chromatography tandem mass spectrometry technique. The assay allows simultaneous quantification of 188 metabolites, including 40 acylcarnitines, 21 amino acids, 21 biogenic amines, level of hexoses, 15 sphingolipids and 90 glycerophospholipids. The kit has been validated according to FDA guidelines and Biocrates company holds a ISO 9001:2008 certification of quality. Stable isotope standards are used for the quantification of biogenic amines and amino acids, using 7-point calibration curve. Acylcarnitines, phospho- and sphingolipids, and hexose were quantified by their relative intensity over the chosen isotopically labeled internal standard. In addition, 3 quality control standards are measured to ensure the normalization of signal intensities during inter-plate measurements. The metabolite concentrations were calculated linearly using a combination of Analyst (ABSciex, Framingham, USA) and MetIDQ (Biocrates Life Sciences AG, Innsbruck, Austria) software. This is done for amino acids and biogenic amines in the LC mode. Due to lack of isotopic standards for lipids and acylcarnitines in the FIA mode the results are classified as semi-quantitative.

Supplementary Table S10.

Limits of detection. The absolute minimum limit of detection (LOD) for a metabolite is largely dependent on the sensitivity of the mass-spectrometer and the ionization of a metabolite. LODs, lower limit of quantification (LLOQ) and upper limit of quantification (ULOQ) for selected metabolites are given in table S5 (FIA) and table S6 (LC). Metabolites marked with an asterisk (*) are isotope corrected.

Analyte		Quality Type (FIA)		Evaluated Quantification		
**MetIDQ Short Name**	**Biochemical Name**	**Valid**	**Semi**	**LOD (µM)**	**LLOQ (µM)**	**ULOQ (µM)**
C0	Carnitine	X		4	5	120
C2	Acetylcarnitine	X		0.15	0.4	35
C4	Bytyrylcarnitine*	X		0.03	0.4	12
C5	Valerylcarnitine*	X		0.04	0.4	12
C16:1	Hexadecenoylcarnitine*		X	0.06		
C18:1	Octadecenoylcarnitine*		X	0.04		
H1	Hexoses	X		20	200	30000

Ac-Orn	Acetyl-ornithine		X	0.15	1	40
Alpha-AAA	Alpha-aminoadipic acid		X	0.3	1	80
Carnosine	Carnosine	X		0.1	0.5	40
Putrescine	Putrescine	X		0.03	0.1	8
Ile	Isoleucine	X		1.5	5	400
Leu	Leucine	X		1.5	50	400
Val	Valine	X		0.5	10	800
PC(16:1/0:0)	Lysophosphatidylcholine acyl C16:1		X	0.07		
PC(C17:0/0:0)	Lysophosphatidylcholine acyl C17:0		X	0.05		
PC aa C32:1	Phosphatidylcholine diacyl C32:1*		X	0.06		
PC aa C32:2	Phosphatidylcholine diacyl C32:2*		X	0.03		
PC aa C34:3	Phosphatidylcholine diacyl C34:3*		X	0.01		
PC aa C34:4	Phosphatidylcholine diacyl C34:4		X	0.006		
PC aa C36:2	Phosphatidylcholine diacyl C36:2*		X	0.15		
PC aa C40:5	Phosphatidylcholine diacyl C40:5*		X	0.04		
PC aa C40:6	Phosphatidylcholine diacyl C40:6*		X	1.2		
PC ae C34:3	Phosphatidylcholine acyl-alkyl C34:3		X	0.015		
PC ae C36:2	Phosphatidylcholine acyl-alkyl C36:2*		X	0.01		
PC ae C38:2	Phosphatidylcholine acyl-alkyl C38:2*		X	0.018		
PC ae C40:6	Phosphatidylcholine acyl-alkyl C40:6*		X	0.025		
SM (OH) C14:1	Hydroxysphingomyeline C14:1*		X	0.025		
SM (OH) C22:1	Hydroxysphingomyeline C22:1*		X	0.015		
SM (OH) C22:2	Hydroxysphingomyeline C22:2*		X	0.01		
SM (OH) C24:1	Hydroxysphingomyeline C24:1*		X	0.01		
SM C24:0	Sphingomyeline C24:0*		X	0.13		
SM C24:1	Sphingomyeline C24:1*		X	0.035		
